# Defect-Mediated Diffusion Pathways in Spodumene Accelerate Lithium Transport

**DOI:** 10.1021/acsmaterialslett.5c00876

**Published:** 2025-09-08

**Authors:** Naman Katyal, Chunhui Li, Martin Kunz, Simon J. Teat, Piotr Zarzycki, Gerbrand Ceder, Michael L. Whittaker

**Affiliations:** † Materials Science Division, 1666 Lawrence Berkeley National Laboratory, Berkeley, California 94720, United States; ‡ Energy Geoscience Division, 1666 Lawrence Berkeley National Laboratory, Berkeley, California 94720, United States; § Advanced Light Source, 1666 Lawrence Berkeley National Laboratory, Berkeley, California 94720, United States; ∥ Department of Materials Science and Engineering, University of California, Berkeley, California 94704, United States

## Abstract

Lithium extraction from naturally occurring α-spodumene is hindered by poor lithium diffusivity, necessitating high-temperature phase transformation to a low-density β polymorph. Although β spodumene exhibits up to 5 orders of magnitude higher lithium-ion diffusivity, both phases have diffusion activation energies between 0.8 and 1 eV, indicating that polymorph density is not the controlling factor over diffusivity. We show that aluminum vacancies facilitate lithium-ion diffusion in α-spodumene by reducing the migration barrier from 2.4 to 0.9 eV. Bond valence site energy and nudged elastic band calculations show a new lithium local minimum site which promotes a one-dimensional percolation network by reducing the lithium intersite distance from 4.5 Å to 2.9 Å. However, aluminum vacancies are energetically unfavorable to percolate through the whole structure, resulting in very low net lithium diffusivity and highlighting the critical role of nonstoichiometric defects in facilitating lithium transport in rigid aluminosilicate structures.

## Graphical abstract

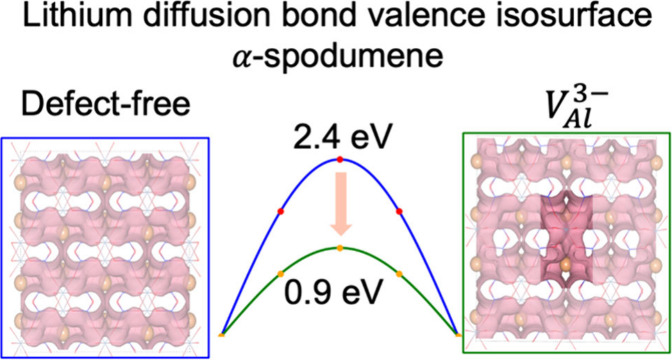

Lithium-ion batteries (LIBs) have become indispensable in portable electronics, transportation, and energy storage (grid) systems.^1–3^ Because of this, up to 95% of global lithium production is projected to be used to manufacture LIBs by 2030, from approximately 60% in 2023.^4^ Pegmatites, containing lithium minerals spodumene, eucryptite, petalite, and lepidolite, are the largest current lithium mineral source^5^ and are distributed more evenly throughout the world compared to salt brines,^6,7^ the other major source of lithium. α-Spodumene (LiAlSi_2_O_6_) is a naturally occurring mineral that crystallizes in the clinopyroxene structure type (space group *C*2/*c*, No. 15). It contains approximately 8 % Li_2_O by weight and has a density of 3.16 g/cm^3^. Aluminum–oxygen octahedra form linear chains parallel to [001] that are connected via common oxygen corners with the characteristic [Si_2_O_4_]^4–^ tetrahedral pyroxene chains, thus forming a three-dimensional (3D) Si–Al-O framework. Lithium atoms occupy interstitial distorted octahedral sites, as shown in Figure 1. α-Spodumene is traditionally heated to 1100 °C to promote phase transition to a low density (2.45 g/cm^3^) tetragonal β polymorph.^8–10^ In this structure, Al and Si atoms occupy the same corner-connected tetrahedral sites while Li atoms occupy half of the interstitial tetrahedral cages.^11,12^ The existence of only corner-sharing aluminosilicate polyhedral structure in β-spodumene compared to edge-sharing aluminum polyhedral structure in α-spodumene significantly affects the lithium mobility in two phases, as recently shown in wide ranging oxide materials.^13^ Higher lithium mobility has also been attributed to greater ionic porosity resulting from differences in the polyhedral network.^14^

**Figure 1:**
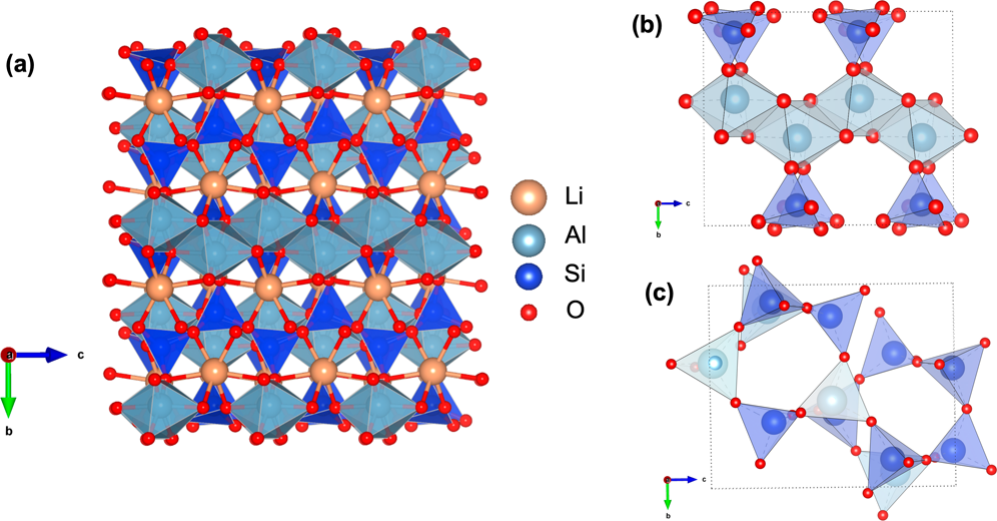
(a) Crystal structure of naturally occurring α-spodumene (LiAlSi_2_O_6_) showing the pyroxene like (Si_2_O_6_)^4–^ chains as well as the edge-sharing aluminum octahedral chains. (b, c) Corner-sharing framework examples: noncorner sharing polyhedral framework in α-spodumene (panel (b)) and corner-sharing polyhedral framework in β spodumene (panel (c)).

A corner-sharing (CS) framework in an oxide crystal structure inherently creates a wide range of distorted environments for Li ions due to high degrees of freedom for positioning nonlithium cation polyhedrons as shown in Figures 1b and 1c for spodumene polymorphs. This high degree of distortion is quantified by continuous symmetry measure (CSM), where CS frameworks show significantly higher and more varied CSM values in comparison to non-CS frameworks. This structural distortion creates low-energy, percolation pathways by minimizing interactions between lithium and nonlithium cations enabling high lithium-ion conductivity.

The α-to-β transformation is often followed by sulfuric acid leaching, in which Li ions are exchanged with protons in the β-spodumene structure^8–10^ and converted to lithium chemical intermediates. β-Spodumene exhibits up to 5 orders of magnitude higher lithium diffusivity than the denser α polymorph,^9,15^ improving lithium yield and reaction rates. However, the high temperature required for the polymorphic transformation makes the overall process energy-inefficient, and methods to facilitate lithium extraction from α-spodumene at lower temperatures would make battery supply chains more sustainable. Understanding lithium diffusion mechanisms in α-spodumene is critical to the development of such processes.

Lithium diffusivity and corresponding activation energies in various spodumene crystals and glasses have been compared through electrical conductivity measurements.^14,16,17^ It was observed that lithium diffusivity in α-spodumene is lower by 3–5 orders of magnitude, compared to spodumene glasses and low-density β and γ polymorphs.^14^ Interestingly, despite the significant differences in diffusivities across these phases, the activation energy for diffusion in both α- and β-spodumene was consistently found to range between 80–100 kJ/mol (0.83–1.04 eV). The slower diffusion in α-spodumene has been attributed to its higher density or lower ionic porosity, which are believed to limit ionic mobility. However, the underlying diffusion mechanism in α-spodumene remains unexplained. Li ions in β-spodumene may occupy either of two equivalent sites that share edges with Al or Si tetrahedra. The Al/Si ratio in β-spodumene can change continuously due to solid solution with abundant β-quartz (SiO_2_), establishing a much broader range of possible coordination environments than that in α-spodumene. We therefore restrict the current study to understanding lithium diffusion in naturally occurring polymorph, which remains heretofore unexplained. The observation of similar activation energies in all phases of spodumene has been previously hypothesized to result from the possible existence of vacancies in α-spodumene, specifically defects associated with aluminum sites,^14,18^ but no detailed investigation into diffusion activation energies and the effect of defects on diffusion has been conducted to date.

In this work, we analyze lithium diffusion pathways and associated energy barriers in both pristine and defective crystals using bond valence^19^ (BV) and bond valence site energy method (BVSE).^20^ BV and BVSE have been applied to determine diffusion pathways and associated energy barriers in various materials by using percolation networks. Percolation networks are composed of percolation pathways that are infinite regions in the crystal lattice through which diffusing ions can move in one, two, or all three dimensions. In each dimension, a percolation pathway has an energy associated with that diffusion (percolation energy barrier), which is calculated using differences in site energies. Identifying percolation pathways with BV­(SE) helps to guide the synthesis of new and novel oxide-ion conductors and allow for screening of fast lithium-ion solid electrolytes, diffusion of monovalent and divalent cations in both crystalline and amorphous structures and anion migration in perovskites,^21–25^ thus enabling high-throughput calculations that bypass the need for computationally expensive density functional theory (DFT) simulations.

Our findings reveal that the lithium percolation energy barrier in the pristine crystal structure exceeds 2.4 eV, which is significantly higher than values (0.83–1.04 eV) inferred from conductivity measurements and thus cannot account for the observed diffusion properties. We demonstrate that percolation energy barriers are consistent with experimental values by systematically enumerating various defect configurations in α-spodumene using BVSE. The reduced activation energies in defect structures result from decreased lithium–lithium interatomic distances from 4.5 Å in the pristine structure to 2.9 Å in defected configurations. This reduction facilitates the formation of a 1D percolation pathway, analogous to olivine-type battery materials, a phenomenon not previously observed in spodumene phases. We validate the BVSE calculations with nudged elastic band (NEB) simulations for both pristine and aluminum defect structures, highlighting the efficacy of BVSE in screening many structures at a fraction of the computational cost of NEB. Finally, we analyze the observed diffusion mechanisms by comparing BV sums from bond valence theory with Bader charge analysis and show that aluminum vacancies create transition states with bond valence charge closer to ideal Li sites that create faster percolation channels.

Five different crystals of α-spodumene, collected from ore concentrate discussed previously,^26^ were measured by synchrotron single-crystal X-ray diffraction (SC-XRD) to obtain the α-spodumene crystal structure used in this work (). Percolation pathways of Li ions in defect-free α-spodumene were calculated using the BVSE method (), as implemented in the BVLain package.^27^ Pathways are visualized as isosurface values of the bond valence site energies in Figure 2a. For defect-free spodumene, the percolation barrier for migration expressed as difference in site energies is 2.38, 2.66, and 3.41 eV in one, two, and three dimensions, respectively. 3-D percolation pathways in defect-free α-spodumene represent the net diffusion of lithium, which is composed of three distinct lithium migration mechanisms. These three unique mechanisms exist between equivalent lithium sites and together form an infinite 3-D percolation network in defect-free α-spodumene. The three unique pathways are represented in Figure 2b in a unit cell from the enclosed region in Figure 2a. These three mechanisms are also shown superimposed on top of the BVSE isosurfaces in . They enable complete 3-D diffusion in a defect-free α-spodumene crystal structure.

**Figure 2:**
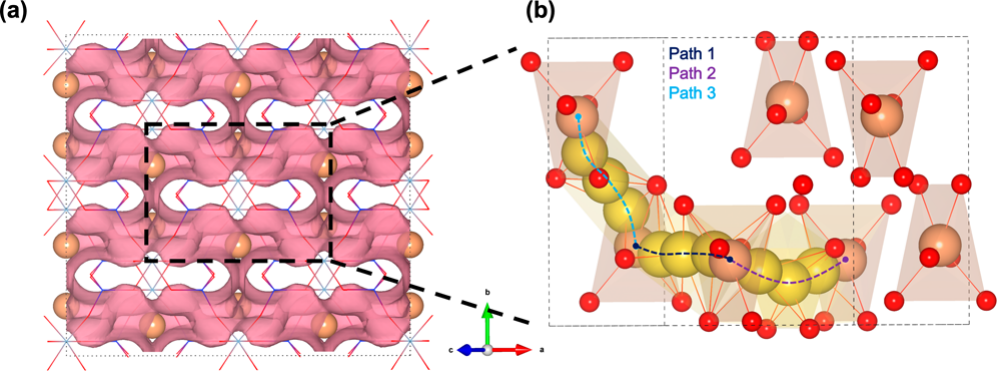
(a) BVSE isosurface of 3D lithium percolation in a defect-free α-spodumene 2 × 2 × 2 supercell. (b) Three unique lithium migration pathways (minimum energy path) from DFT-NEB idealized in a defect-free α-spodumene unit cell from the region enclosed in a black box in panel (a). Color scheme: orange (Li), yellow (Li atoms along the minimum energy path).

Lithium diffusion energetics were further analyzed using DFT-NEB simulations (). A distorted octahedron with an energy barrier of 1.77 eV is the transition state in Path 1, with Li–O bond lengths of 2 × 1.87 Å, 2 × 2.17 Å, and 2 × 2.41 Å. A tetrahedral transition state with a 2.30 eV barrier forms four oxygen bonds 1.83 Å in length in Path 2. The Path 3 transition state with a barrier of 2.00 eV has a square planar geometry with two 1.74 Å oxygen bonds and two 2.16 Å bonds. The minimum energy path for lithium migration and the transition state geometry for each percolation pathway is shown in . Since the lithium 3-D percolation energy barriers and activation energy of migration paths are higher than experimental diffusion barriers, it is unlikely that experimental Li diffusivities correspond to defect-free α-spodumene.

Three types of α-spodumene unit cell defects were investigated using the BVSE method: Type 1, V_Al_^3–^; Type 2, Al_Si_^1+^ + Si_Al_^1–^; and Type 3, Si_Al_^1+^ + V_Li_^1–^. A total of nine defect structures were studied in this work: one Type 1 (aluminum vacancy), four Type 2 (silicon–aluminum swap), and four Type 3 (silicon substitution for aluminum and lithium). The nine defect structures in the unit cell are shown in . These defects in α-spodumene maintain charge neutrality in the simulation box and fall within the observed up to 0.10 atom-per-formula-unit of missing aluminum site vacancy in α-spodumene crystals. We did not consider other defects like V_Si_^4–^(silicon vacancy), Al_Si_^1–^ + Li_i_^1+^ (Li–Al pair substitution of Si) for which there is no experimental evidence in spodumene, nor did we consider heteroatom substitutions for which there is no observed correlation with lithium diffusivity.^18^ Each defect structure geometry was first optimized with the CHGNET^28^ interatomic potential that was fine-tuned with DFT-optimized structures of spodumene prior to BVSE analysis. Geometry optimization of defect structures is important to allow relaxation of the bond lengths to achieve a local minimum-energy arrangement compared to pristine structure prior to bond valence analysis.

The lithium percolation energy barriers for all nine structures from the BVSE method are tabulated in Table 1. The lowest percolation barrier of 0.9 eV for lithium migration was obtained with V_Al_^3–^(neutral aluminum vacancy) due to the formation of a new pathway. The BVSE isosurface for V_Al_^3–^, as shown in Figure 3a and , spans the *a*–*c* plane that connects to the nearest Li ions around V_Al_^3–^. Relaxation near the vacancy reduced the nearest lithium–lithium distance to 2.9 Å, compared to 4.5 Å in the defect-free structure, due to the emergence of a new lithium site created near V_Al_^3–^. This new site establishes a link between the pathways for the two mechanisms (see Figures 3a and 3b, as well as ). Similar to pristine α-spodumene, migration barriers between equivalent Li sites were calculated using NEB simulations for the V_Al_^3–^ structure using DFT, and the model structure is shown in . All calculated energy barriers for lithium migrations in V_Al_^3–^are tabulated in . The two lowest energy barriers for lithium migration from NEB simulations are 1.12 and 0.93 eV through the newly created lithium site adjacent to the aluminum vacancy. The minimum energy path for lithium migration is shown superimposed on the BVSE isosurface in . Therefore, the 1D percolation energy barrier from BVSE and activation energy from NEB calculations for the fastest lithium migration pathway in V_Al_^3–^ α-spodumene are in good agreement with experimentally measured activation energies. There is a small reduction in the energy barrier for Types 2 and 3 defects (Table 1), relative to the pristine structure, but the barriers are significantly higher than those for the Type 1 defect.

**Table 1:** 1D, 2D, and 3D Percolation Energy Barriers of Lithium Migration in Different Spodumene Structures from BVSE

	V_Al_^3–^ (eV)	Al_Si_^1+^ + Si_Al_^1–^ (eV)	Si_Al_^1+^ + V_Li_^1–^ (eV)	pristine (eV)
Percolation Path 1D	0.88	2.08, 2.1, 2.06, 2.19	2.16, 2.65, 2.17, 2.2	2.38
Percolation Path 2D	2.89	2.74, 2.96, 2.85, 3.0	2.78, 2.75, 2.54, 2.62	2.66
Percolation Path 3D	4.26	3.35, 3.16, 3.14, 3.28	3.35, 3.16, 3.14, 3.28	3.41

**Figure 3:**
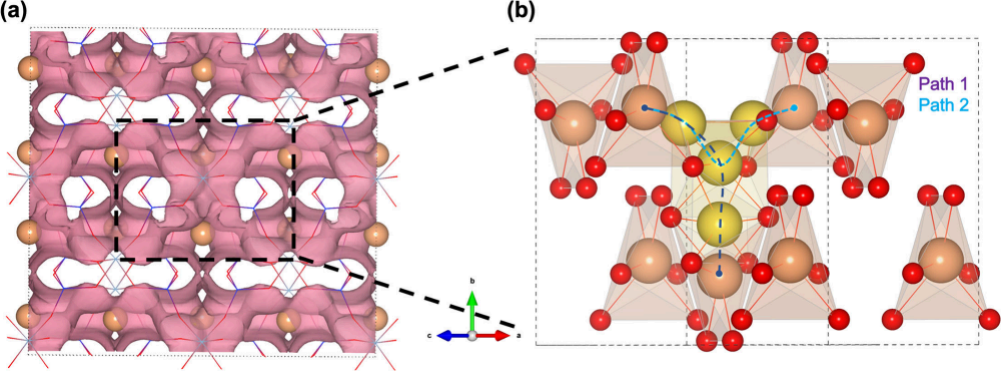
(a) Isosurface of 3D percolation pathways from BVSE method in a V_Al_^3–^ defect α-spodumene 2 × 2 × 2 supercell. (b) Two different and unique lithium migration pathways (minimum energy path) from DFT-NEB idealized in an α-spodumene unit cell from region enclosed in black box in panel (a). Color scheme: orange (Li), yellow (Li atoms along the minimum energy path).

Transition states for Li migration in α-spodumene exhibit shorter bonds and lower lithium charges than equilibrium lattice sites. Bader charge from DFT-NEB was directly correlated to BV sum for both equilibrium and transition sites, allowing D-Map contours () to be interpreted as iso-potential surfaces.^19^ D-Maps for lithium diffusion in the *a*–*c* plane for the defect free α-spodumene structure are shown in Figure 4a with the corresponding lattice plane slices marked in Figure 4b. Maps perpendicular to the *a*–*c* plane are shown in at different isosurface values. The bond valence sums from D-Map and the corresponding Bader charge from DFT are tabulated in . Bond valence sums of 1.14 v.u. correspond to the ideal lithium site on the *a*–*c* plane, which is shown by an isosurface value of 1 in Figure 4a marked with white crosses. The D-Map shows the diffusion pathway for the lithium along the isosurface value of 0.85, which corresponds to a bond valence sum of 1.45 v.u. at the transition state and BVSE migration barrier of 2.39 eV for Path 1. The transition state octahedron has smaller bond lengths on average compared to the initial state which leads to a higher bond valence sum. The Bader charge on the initial and transition state from NEB simulations for this migration pathway is 0.89 and 0.85. Similarly, a smaller Bader charge on Li ions at the transition state also indicates more electron density on lithium which directly aligns with more valence on lithium from bond valence theory. The lithium diffusion for Path 2 is observed at a D-Map isosurface value of 0.75, which corresponds to a bond valence sum of 1.74 v.u. at the transition state (Figure 4a) and BVSE migration barrier of 3.41 eV. Therefore, qualitatively, the bigger the change in bond valence sum between initial and transition state, the bigger is the energy barrier predicted from the BVSE method. Specifically, between the three percolation energy barriers, the change in bond valence sums between the smallest and largest energy barrier is 0.31 v.u. and 0.60 v.u., respectively.

**Figure 4:**
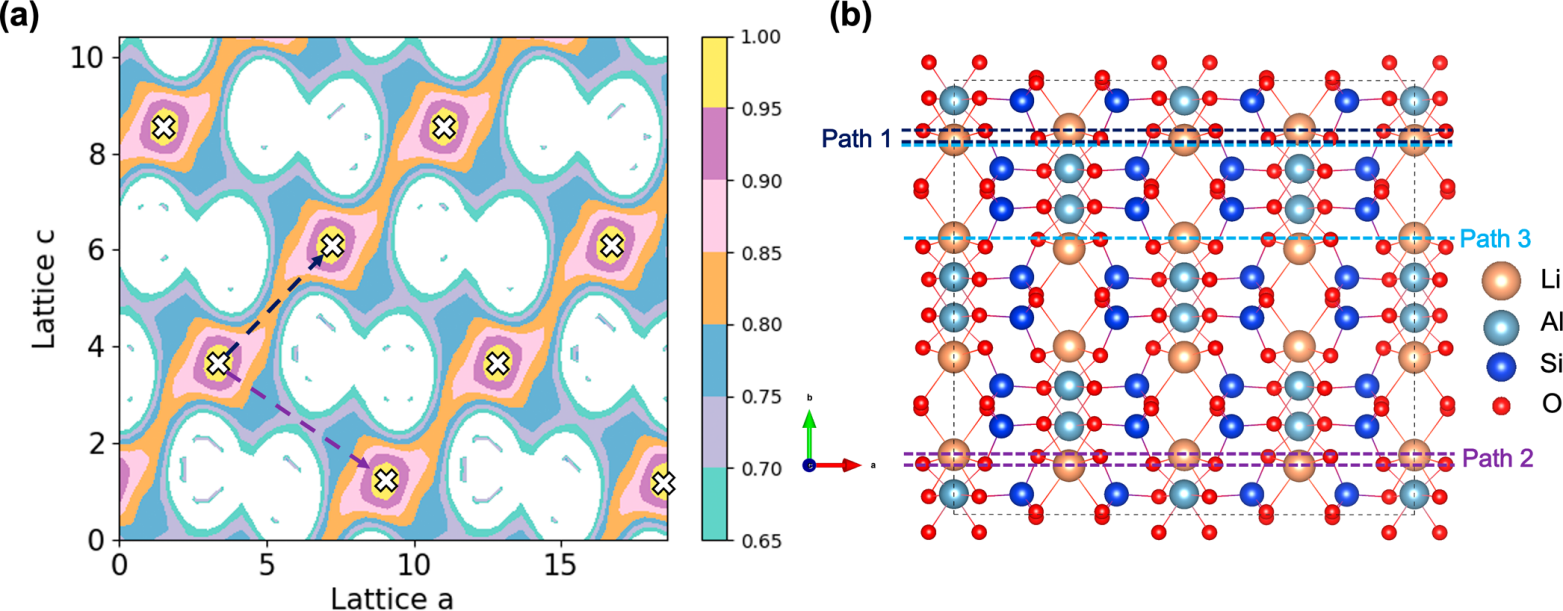
(a) D-Map of Path 1 and 2 diffusion pathways of lithium in defect free α-spodumene with pathways marked as dashed lines and lithium sites marked as white crosses. (b) The planar cross-section of three Paths in a 2 × 2 × 2 supercell of α-spodumene used to calculate D-Maps in panel (a) and .

D-Maps for lithium diffusion and bond valence sums at the transition states in V_Al_^3–^ α-spodumene were compared to those of the transition state in the defect-free structure. The D-Map of lithium diffusion for the (V_Al_^3–^) aluminum defect α-spodumene structure is shown in Figure 5a for Path 2 (Figures 3b). The slice of the supercell used for the D-Map evaluation is depicted in Figure 5b. The ideal lithium sites in the supercell are marked with red crosses, and the new minimum created due the aluminum defect is marked with a white cross. Due to the new lithium site, a connecting pathway is created at an isosurface of 0.80 between the nearest Li sites which did not exist in the D-Map of pristine α-spodumene (Figure 4a). It can be clearly seen that the diffusion pathway between the lithium sites (red) passes through the new lithium site which thus reduces the nearest lithium–lithium distance required for percolation. For this migration path, the bond valence sum of lithium in the initial and transition state is 1.18 v.u. and 1.29 v.u, respectively. The bond valence sum of lithium in the initial state in this case is higher than defect free structure (1.18 v.u. vs 1.14 v.u.) because there is an aluminum ion missing around the lithium and the surrounding oxygen ions therefore form stronger bonds (high bond valence) with lithium to compensate for the missing Al charge. The change in the bond valence sum along this migration path is 0.11 v.u., which is smaller than the change in the defect-free structure of 0.31 v.u., which qualitatively indicates a reduction in activation energy. The D-Map of lithium diffusion along only the slice where an aluminum vacancy is present is shown in to visualize the bond valence sums around the vacancy site and the connecting Li atoms. Furthermore, this diffusion pathway connects to the other Li atoms in the plane at an isosurface value of 0.75, which, in addition, enables lithium percolation throughout the plane, similar to the one shown in Figure 4a. Therefore, bond valence sums are a meaningful representation for the charge of mobile atoms which can be correlated with energy barriers. The excellent agreement of the migration energy barrier prediction from BVSE and calculated from DFT-NEB in aluminum vacancy α-spodumene is attributed to the negligible changes in the local environment (Si–O and Al–O) around the diffusing Li ion ().

**Figure 5:**
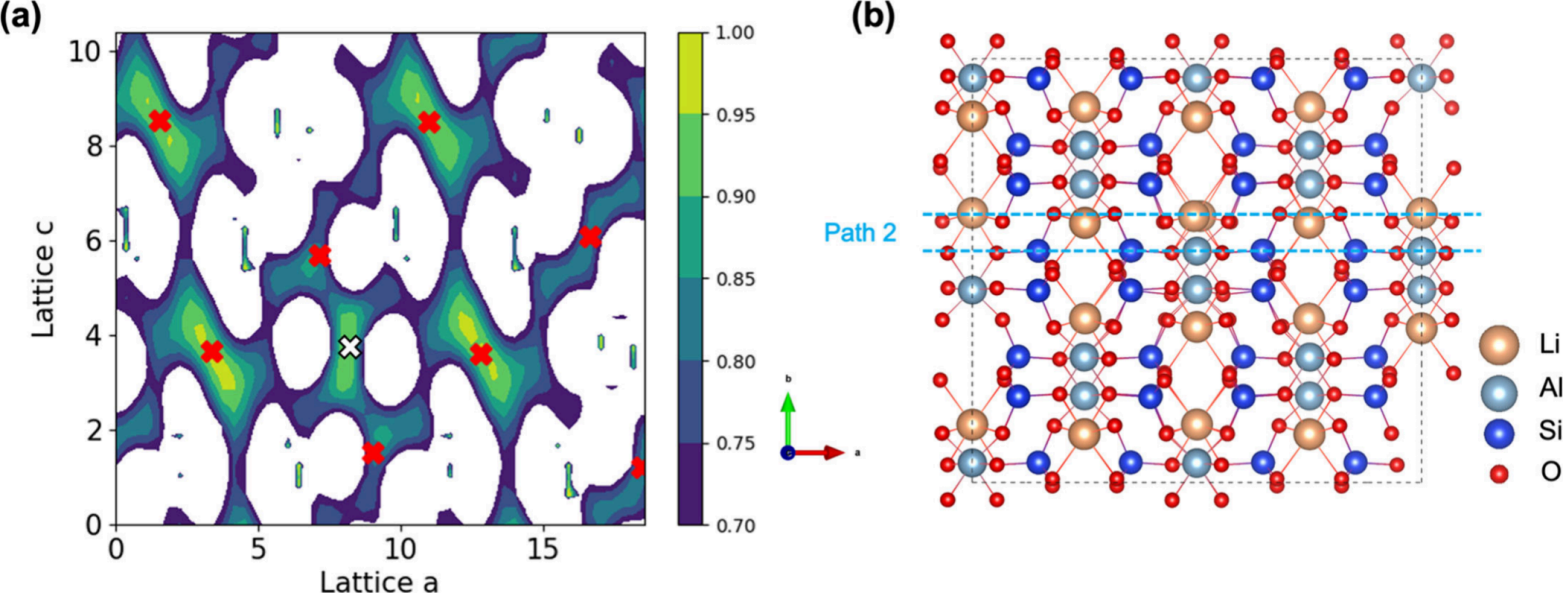
(a) D-Map of lithium diffusion pathway in V_Al_^3–^ defect α-spodumene for Li sites marked in red crosses via a new lithium site marked as a white cross. (b) The planar cross-section of a V_Al_^3–^ defect α-spodumene 2 × 2 × 2 supercell structure used for D-Map calculation in panel (a).

The reduction in the lithium migration energy barrier is due to creation of a new stable lithium site around the aluminum vacancy. A new stable lithium site is created near the aluminum vacancy site that connects to the nearest percolating lithium sites at a distance of 2.98 Å, compared to the pristine structure where the Li–Li interatomic separation was 4.5 Å. The reduction in Li–Li interatomic distance along the minimum energy path requires a smaller change in bond valence sum (0.11 v.u.), compared to pristine material (0.31 v.u.), which reduces the migration energy barrier (). However, percolating V_Al_^3–^ is required for lithium conduction to be equal to 0.125 Al per formula unit. These defects must exist from geological formation conditions which exist in the presence of cations such as Fe, Mg, and Na in the spodumene concentrate from feldspar and mica.

## Supplementary Material







## Data Availability

The scripts used in this work for D-Map and BVSE analysis as well as bond valence parameter file can be accessed via github (https://github.com/n2018k/BVSE-Manuscript).

## Notes

Single Crystal X-Ray Diffraction (SCXRD) (Supplementary Note 1); Bond Valence (BV) and Bond Valence Site Energy (BVSE) Method (Supplementary Note 2); Density Functional Theory Calculations (Supplementary Note 3); α- Spodumene Crystal Structure (Supplementary Note 4); Agreement between BVSE and DFT-NEB (Supplementary Note 5); Similarity to other 1-D lithium ion conductors (Supplementary Note 6); Supplementary figures and tables discussed in the main text (PDF)
The bond valence analysis for five single-crystal samples and SC-XRD summary (XLSX)
Fitting result of SC-XRD of all spodumene samples used in this work, bond valence analysis of spodumene samples; lithium percolation pathway from BVSE, D-Map of pristine and defect α- spodumene, NEB simulations minimum energy path and barriers from DFT simulations, analysis of bond valence charge and Bader of pristine and defect α- spodumene (ZIP)
