# Supplementary material for: Defect-Mediated Diffusion Pathways in Spodumene Accelerate Lithium Transport
Source: ACS Mater Lett. 2025 Sep 8;7(10):3388–93. doi: 10.1021/acsmaterialslett.5c00876 (PMC12505375; doi:10.1021/acsmaterialslett.5c00876)
Supplement: Supplementary file 1 [file tz5c00876_si_001.pdf]

Defect-mediated diffusion pathways in spodumene accelerate lithium transport

Naman Katyal[1], Chunhui Li[1], Martin Kunz[3], Simon J. Teat[3], Piotr Zarzycki[2], Gerbrand Ceder[1,4], Michael L. Whittaker[1,2]

[1]Materials Science Division, Lawrence Berkeley National Laboratory, Berkeley, CA 94720

[2]Energy Geoscience Division, Lawrence Berkeley National Laboratory, Berkeley, CA 94720

[3]Advanced Light Source, Lawrence Berkeley National Laboratory, Berkeley, CA 94720

[4]Department of Materials Science and Engineering, University of California, Berkeley, CA 94704

Supplementary Note 1: Single Crystal X-Ray Diffraction (SCXRD)

Single crystals were selected and mounted on MiTeGen® loops with epoxy resin on a Bruker D8 diffractometer equipped with a PHOTONII CMOS detector and Oxford Cryosystems Cryostream 800 plus, on Beamline 12.2.1 of the Advanced Light Source at LBNL. A sphere of data was collected at 100K, using Bruker APEX3 software in shutterless mode with ω rotations at fixed φ values at a wavelength of λ= 0.7288Å selected with a double crystal Silicon [111] monochromator. The intensity data were integrated, and correction applied with SAINT v8.40b. Absorption correction and additional processing were done using SADABS 2016/2. Dispersion

corrections appropriate for this wavelength were calculated using the Brennan method in XDIP with in WinGX. The structures were solved with a dual space method with SHELXT 2018/2 and refined using SHELXL 2019/2.

Supplementary Note 2: Bond Valence (BV) and Bond Valence Site Energy (BVSE) Method

The bond valence sums for all the structures were calculated using the available bond valence parameters[1]. The bond lengths for each sample were used to calculate the bond valence sum (observed valence) for each unique ion in a given structure using bond valence parameters. In bond valence theory, contribution of each atom to a bond is called bond valence which quantifies strength/attraction between two atoms. Bond valence is related to bond length (R_{ij}) as:

$$s_{ij} = \exp(\frac{R_0 - R_{ij}}{b})$$

where, R_0 and b are empirical bond valence parameters determined empirically for each atom pair and are chosen such that sum of bond valence is approximately close to atomic valence. Physically, R_0 is the bond length of unit bond valence while b measures softness of the atomic cores. The bond valence parameters R_0 and b for each cation-anion bond pair are tabulated in Table SI-5 which were calculated as an average of all available R_0 and b values for each pair of cation-anion bond. Network bond valences are calculated for the spodumene structure based on the two network equations[2]:

Network Equation 1: the sum of the bond valences incident on an atom is equal to the valence of the atom *i* with its coordinating atoms *j*.

$$V_i = \sum_j s_{ij}$$

Network Equation 2: Considering that all bonds are directed from j atoms to i atoms, in a bipartite network, the sum of bond valences is zero around any closed loop.

$$\sum_{loop} s_{ij} = 0$$

The bond network of spodumene structure is tabulated in Table SI-1. There is one unique Li and one unique Al atom, two unique Si atoms and 6 different oxygen atoms forming the spodumene $LiAlSi_2O_6$ structure as shown in Figure SI-1. In Table SI-1, the row/column Q demonstrates the valence of each atom while the entries in all other cells indicate the number of bonds between each atom type. The bond lengths, observed bond valence, network bond valence and network bond length for each ion pair for all 5 samples is attached as Supporting Information file.

The D-Maps from bond valence sums for lithium diffusion pathways and transition state analysis were calculated using the methodology given elsewhere[3]. D-Maps are calculated as valence maps in the structure by summing up bond valence sums of a lithium atom at an arbitrary point in the crystal. By moving the arbitrary point over a grid of points throughout the unit cell, a D-Map is obtained and plotted as planar cross sections or slices of the unit cell. At local minima in the unit cell, the values in the D-Map will be the ideal valence of the lithium. The values on D-Map are plotted using the equation below:

$$D = \left(\sum_{j} {s_{ij}}/{V_i}\right)^{-N}$$

where s_{ij} is the bond valence of the lithium atom i with its neighbor j at an arbitrary point, V_i is the valence of the lithium atom, and $N = 1$. All D-Maps were plotted using matplotlib module in python and representative scripts for calculating bond valence sums and corresponding graphs have been attached for reproducing the results.

The global instability index (G) is defined as first proposed by Brown[4], which is defined as root mean squared difference between the experimental and network bond valence sum of each atom in the structure, calculated using equation below:

$$G = \left(\frac{\sum_i \left\{ \left(V_i - \sum_j s_{ij} \right)^2 \right\}}{n} \right)^{1/2}$$

where G is the global instability index, V_i is the valence of atom, s_{ij} is the bond valence of atom with individual neighbor, n is the total number of unique atoms in the unit cell.

The lithium diffusion pathway using the bond valence site energy method (BVSE) was calculated for the spodumene structure with and without defects using a BVLain[5] module and the corresponding isosurfaces were plotted using the VESTA package[6]. In this method, bond valence parameters are used to represent energy for every point/site in the structure by calculating a Morse term and a Coulomb term. Finally, these site energies are represented as an isosurface similar to the bond valence sum, depicting percolating diffusion pathways and predicting energy barriers in many inorganic materials. All geometry optimizations for defect structures prior to BVSE analysis was performed using the CHGNET[7] machine learning potential fine-tuned for spodumene configurations with density functional theory (DFT) calculations performed with the Vienna ab-initio simulation package (VASP)[7]. The details for VASP settings are explained in the DFT sections.

Supplementary Note 3: Density Functional Theory Calculations

The electronic structure for spodumene was optimized using density functional theory as implemented in the Vienna ab-initio simulation package (VASP)[8,9]. The semi local density functional (SCAN) level of the generalized gradient approximation was used for all calculations in this work[10]. The projector augmented wave method was used to model core electrons; the kinetic energy cutoff for plane waves was chosen as 500 eV to describe valence electrons and reciprocal space was sampled with mesh density[11] of 3*4*6. PAW Li, O, Si, and Al pseudopotentials were used for all calculations in this work. The electronic and ionic optimization convergence was chosen as 10^{-6} eV and maximum force on all atoms was less than 0.01 eV/Å, respectively. The lithium diffusion pathway in the pristine and defect spodumene crystal structure was calculated as a vacancy mediated mechanism using the nudged elastic band method (NEB)[12] on a 2*2*2 supercell with 5 intermediate images to minimize vacancy self-interactions. Finally, a Bader charge analysis was performed using the Bader code available from all electron charge densities derived from VASP after geometry optimization for minima and transition states[13]. All diffusion pathways from NEB were visualized using the VESTA package.

Supplementary Note 4: Alpha Spodumene Crystal Structure

SCXRD of the spodumene crystals from the concentrate was performed to quantify all bond lengths in the structure for bond valence analysis. The results for individual sample are attached as Supporting Information files. The average unit cell parameters of the crystal were a = 9.464Å, b = 8.382Å, c = 5.222Å and beta = 110.242° and indexed to the C2/c spacegroup (no. 15). The standard deviation in the unit cell parameters were 0.011, 0.012, 0.008 and 0.037 respectively indicating homogeneity in all samples. The average bond lengths for Al-O, Li-O and Si-O in the single crystals is Tabulated in Table SI-2, all of which are consistent among the five replicates. Cell parameters and refinement details are summarized in Table SI-4. Observed and network bond valence sums for the spodumene crystal are in good agreement for Si^{4+} and Al^{3+} cations as shown in Table SI-6-8 (Supplementary Note 2). The average bond length, network bond valence, (in bond length and bond valence) are

equivalent within standard deviations for the observed and ideal (i.e. as predicted by the BV network equations (Supplementary Note 2) Si-O and Al-O bonds. This indicates that silicon and aluminum atoms are situated in the centers of nearly ideal polyhedra. However, observed Li-O bond lengths and valences are substantially different from the predicted network bond lengths and valences. The average bond lengths from SC-XRD and network bond valences are similar (2.20Å vs. 2.18Å), but variations in individual Li-O bond lengths contribute to the difference in observed and predicted valence sums as shown in Table SI-3.

The mismatch between the observed Li-O bonds and the predicted values is likely due to geometric frustrations when mapping the ideal bond network into three-dimensional space ('steric strain')[14]. The Si, Al-oxide framework creates distorted octahedral interstitial sites that are slightly too large for Li cations to occupy with unstrained bonds. This can be partially alleviated by the off-center-position of the Li-atom in its site. Lattice site displacements that lead to valence sum deviations can be quantified using the global instability index (G)[4] which is defined as the root mean squared difference between the experimental and network bond valence sums of each atom in the structure (see Supplementary Note 2 for equation). The G value of spodumene crystals from SC-XRD is 0.098, falling in the middle of the proposed stability range $0.05 \leq G \leq 0.20$. This is consistent with calorimetric measurements of spodumene formation energies and DFT calculations showing that alpha spodumene is stable, and the lowest energy phase at this composition[15,16]. However, G=0.1 implies systematic bond length distortion.

Supplementary Note 5: Agreement between BVSE and DFT-NEB

Specifically, the Si-O and Al-O bond lengths do not change significantly around diffusing lithium ion in the first nearest neighbors. A maximum change in Si-O/Al-O bonds of 1.2% (0.02Å) in the aluminum vacancy structure was observed from DFT-NEB between the initial and transition state configuration whereas a maximum change for the same bonds of 3% (0.06Å) to the nearest oxygen atoms was observed in the defect free alpha

spodumene structure between the initial and transition state configuration. The significantly larger change in bond lengths predicted from DFT-NEB in the defect free alpha spodumene structure should translate to a bigger change in the BVSE analysis at the transition state around the lithium ion. However, this cannot be observed with the BVSE method since it is a static method where dynamic changes in bond lengths are not modelled. Site energies are calculated for the entire structure using the atomic positions of the ground state structure, but atomic positions at the transition states are usually significantly different. Therefore, there is a significant discrepancy in the activation energy predictions from BVSE and calculated from DFT-NEB in the defect-free structure but good agreement in the aluminum vacancy structure.

The 1D, 2D and 3D percolation in defect free alpha spodumene predicted with BVSE corresponds to an energy barrier of 2.38 eV, 2.66 eV and 3.41 eV, respectively. The bond valence sums change from 1.14 v.u. to 1.45 v.u., 1.14 v.u to 1.60 v.u., and 1.14 v.u. to 1.74 v.u. along three unique lithium migration pathway forming the 3D percolation network. The calculated activation energies from BVSE method (and DFT-NEB) are significantly higher than the observed diffusion activation energy of lithium from conductivity measurements.

Supplementary Note 6: Similarity to other 1-D lithium ion conductors

A 1D percolation energy barrier of 0.9 eV was found to be the smallest for the structure with aluminum ion defects using BVSE and confirmed by DFT-NEB calculations. Since 1D percolation energy barriers from the BVSE method are in good agreement with measured activation energies from conductivity measurements in alpha spodumene, this strongly suggests that lithium diffusion observed in alpha spodumene is primarily 1D, similar to battery materials like $LiFePO_4$[17]. The nearest Li-Li interatomic distance along the percolating b-axis in $LiFePO_4$ is also 2.98Å, with a diffusion barrier of 0.6 eV[18].

Table SI-1: Bond Network in alpha spodumene crystal structure. Color scheme for each element in accordance with atom type in Figure SI-1.

Alpha	Q	Li	Al	Si1	Si2
Q		1	3	4	4
O3_1	2	1	2	1	0
O3_2	2	1	2	0	1
O4_1	2	1	0	1	1
O4_2	2	1	0	1	1
O5_1	2	1	1	1	0
O5_2	2	1	1	0	1

Table SI-2: Average bond lengths (Å) with standard deviations and bond valence sums (v.u.) for each ion-pair and ion in 5 spodumene samples in this work from SC-XRD measurements.

Sample	Si-O (avg)	Al-O (avg)	Li-O (avg)	BVS(Si)	BVS(Al)	BVS(Li)
1	1.6212±0.022	1.9212±0.080	2.2086±0.086	4.07	2.94	0.93
2	1.6212±0.023	1.9198±0.080	2.2072±0.088	4.07	2.95	0.93
3	1.6216±0.023	1.9207±0.080	2.2086±0.084	4.07	2.94	0.93
4	1.6166±0.022	1.9149±0.080	2.2003±0.086	4.12	2.99	0.94
5	1.6223±0.022	1.9205±0.080	2.2084±0.086	4.06	2.95	0.88

Table SI-3: Measured and observed bond lengths and overserved and network bond valence sums for Li-O bonds in Sample 5.

**Li-O**	*Bond Length (Å)*	**Observed Valence (v.u.)**	Network Bond Valence **(v.u.)**	***Network Bond Length(Å)***
**O3_1/O3_2**	2.097	0.180	0.125	2.283
**O4_1/O4_2**	2.239	0.136	0.1	2.397
**O5_1/O5_2**	2.279	0.126	0.275	1.881
**Summed**		0.885	1	

Table SI-4: Single crystal x-ray diffraction results of five spodumene samples in this study.

	**Sample 1**	**Sample 2**	**Sample 3**	**Sample 4**	**Sample 5**
**Temperature (C)**	-173	-173	-173	-173	-173
**Crystal System**	monoclinic	monoclinic	monoclinic	monoclinic	monoclinic
**Space Group**	C2/c	C2/c	C2/c	C2/c	C2/c
**a (Å)**	9.4702(10)	9.4667(9)	9.468(9)	9.444(13)	9.4556(13)
**b (Å)**	8.3885(8)	8.3854(8)	8.386(8)	8.3606(11)	8.3753(11)
**c (Å)**	5.2295(5)	5.222(5)	5.2262(5)	5.209(7)	5.2213(7)
**beta**	110.281(4)	110.211(3)	110.196(3)	110.257(5)	110.272(5)
**Volume (Å^3)**	389.68	389.02	389.44	385.87	387.88

**Max. 2-theta**	72.95	73.14	73.13	71.27	70.95
**Limiting indices**	[-15,h,15]	[-15,h,15]	[-15,h,15]	[-15,h,14]	[-15,h,14]
	[-13,k,13]	[-13,k,13]	[-13,k,13]	[-13,k,13]	[-13,k,13]
	[-8,l,7]	[-8,l,8]	[-8,l,8]	[-8,l,8]	[-7,l,8]
**Data/restraints/parameters**	849/0/47	865/0/47	870/0/47	825/0/47	822/0/47
**Goodness-of-fit on F^2**	1.139	1.21	1.153	1.109	1.176
**R1**	0.037347	0.021236	0.025914	0.050246	0.0385
**wR2**	0.116199	0.071526	0.076721	0.146241	0.1244

Table SI-5: Bond Valence parameter for each ion pair used in this work.

**System**	***R0*** **(**Å)	**b (**Å)
**Li-O**	1.223	0.51
**Si-O**	1.6275	0.3747
**Al-O**	1.643	0.38

Table SI-6: Observed and network aluminum valence sum results for Sample 5.

	Bond Length (Å)	**Observed Valence**	Network Bond Valence	***Network Bond Length(Å)***

Al-O				
**O3_1**	1.9441	0.452	0.45	1.946
**O3_2**	1.9909	0.400	0.45	1.946
**O5_1/O5_2**	1.8186	0.629	0.6	1.837
**Summed**		2.965	3	

Table SI-7: Observed and network silicon valence sum results for Sample 5.

**Si-O**	*Bond Length (Å)*	**Observed Valence**	Network Bond Valence	***Network Bond Length(Å)***
**O3_2**	1.6397	0.967	0.975	1.636
**O4_1**	1.6241	1.009	0.95	1.646
**O4_2**	1.6288	0.996	0.95	1.646
**O5_2**	1.5856	1.118	1.125	1.583
**Summed**		4.09	4	

Table SI-8: Observed and network oxygen valence sum (3 unique oxygens from bond network table in Table SI-1) results for Sample 5.

**O3**	*Bond Length (Å)*	**Observed Valence**	Network Bond Valence	***Network Bond Length(Å)***
**Li**	2.100	0.178	0.125	2.283
**Si**	1.6429	0.959	0.975	1.636

**Al**	1.9459	0.450	0.45	1.946
**Al**	1.9941	0.396	0.45	2.283
**Summed**		1.983	2	

**O4**	*Bond Length (Å)*	**Observed Valence**	Network Bond Valence	***Network Bond Length(Å)***
**Li**	2.2435	0.135	0.1	2.397
**Si**	1.626	1.004	0.95	1.646
**Si**	1.6313	0.989	0.95	1.646
**Summed**		2.128	2	

**O5**	*Bond Length (Å)*	**Observed Valence**	Network Bond Valence	***Network Bond Length(Å)***
**Li**	2.281	0.125	0.275	1.881
**Si**	1.588	1.108	1.125	1.583
**Al**	1.821	0.625	0.6	1.837
**Summed**		1.858	2	

Table SI-9: All DFT-NEB lithium diffusion energy barriers in single aluminum vacancy $LiAlSi_2O_6$ 2*2*2 supercell for the structure show in **Figure SI-9** below.

Diffusion Pathway	Energy Barrier (eV)
16 → 15	2.23
8→7	0.93
8→27	1.85
8→31	2.07
8→13	0.85
8→16	2.0
18→14	1.8
18→17	2.2
28→27	2.24

Table SI-10: Bond valence charge and Bader charge at the initial and transition state of each diffusion pathway in the pristine and defect alpha spodumene structure.

Paths	Initial State BV charge	Transition State BV charge	DFT Bader Charge Initial State	DFT Bader Charge Transition State	DFT-NEB Barrier
Defect Free Path 1	1.14	1.45	0.89	0.85	1.77 eV
Defect Free Path 2	1.14	1.74	0.89	0.82	2.3 eV
Defect Free Path 3	1.14	1.60	0.89	0.85	2.0 eV
Al vacancy Path 2	1.18 (BV Sum at new minimum: 1.30)	1.29	0.89	0.87	1.12 eV
Al vacancy Path 1	1.18 (BV Sum at new	1.57	0.89	0.87	0.9 eV

	minimum: 1.30)				

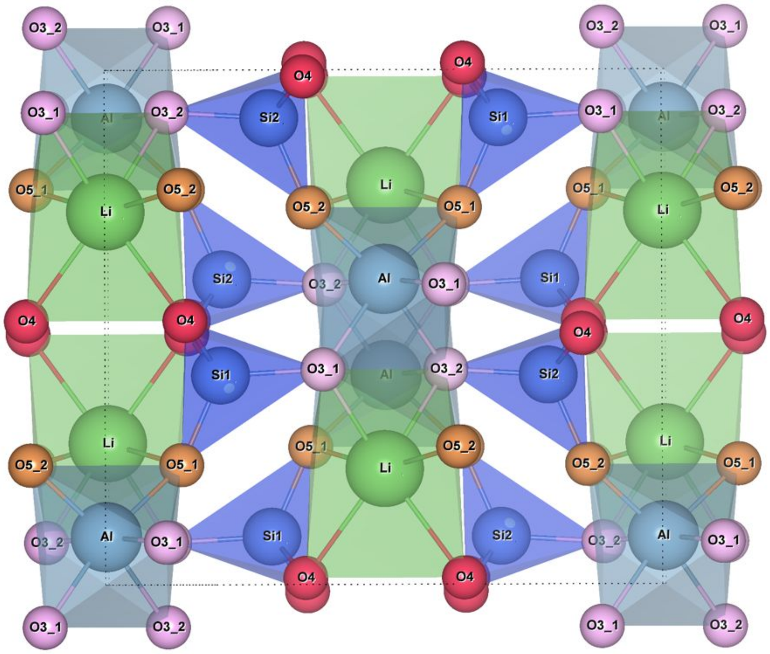

Figure SI-1: Crystal structure of alpha spodumene with unique atom types color coded independently for bond network analysis in Table SI-1.

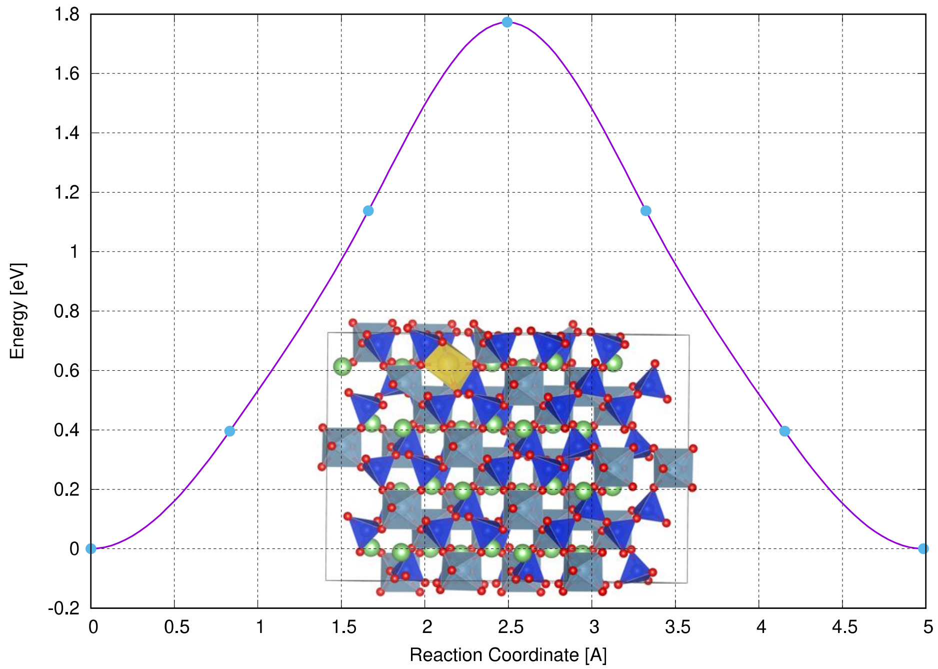

Figure SI-2: Minimum energy path for lithium diffusion mechanism from DFT-NEB for energy barrier 1.77 eV (Path 1). Lithium at the transition state is shown in yellow color octahedron. Color scheme: blue (Si), green(Li), gray(Al) and red(O).

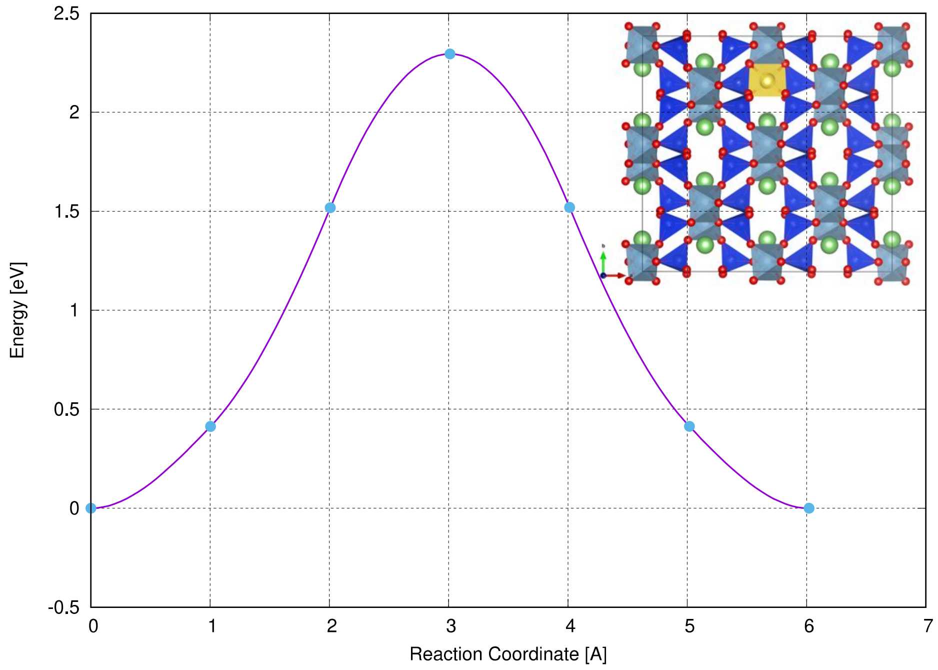

Figure SI-3: Minimum energy path for lithium diffusion mechanism from DFT-NEB for highest 1D energy barrier 2.3 eV (Path 2). Lithium at the transition state is shown in yellow color tetrahedron. Color scheme: blue (Si), green(Li), gray(Al) and red(O).

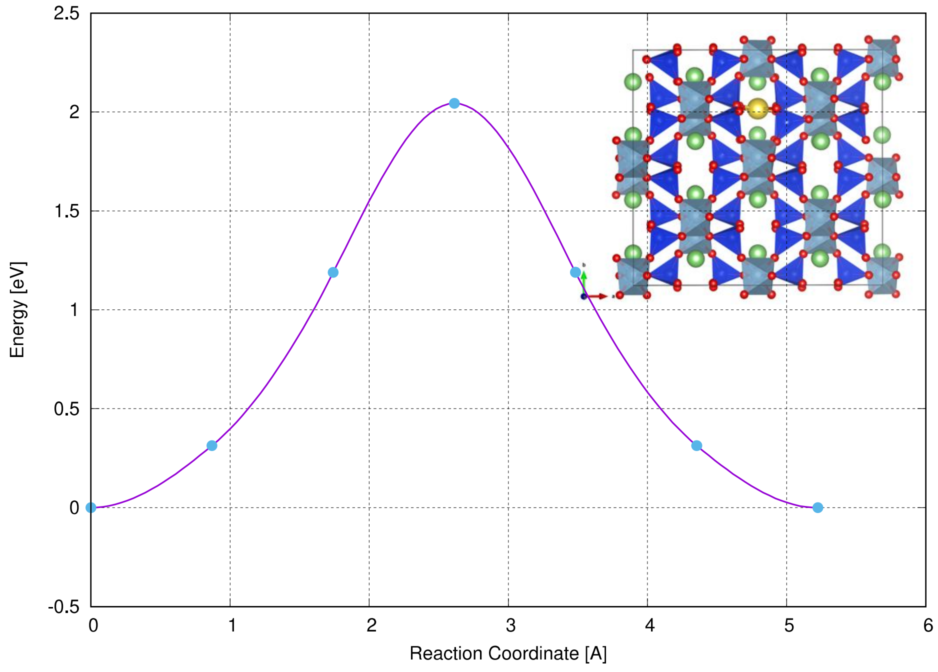

Figure SI-4: Minimum energy path for lithium diffusion mechanism from DFT-NEB for intermediate 1D energy barrier 2.0 eV (Path 3). Lithium at the transition state is shown in yellow color square planar. Color scheme: blue (Si), green(Li), gray(Al) and red(O).

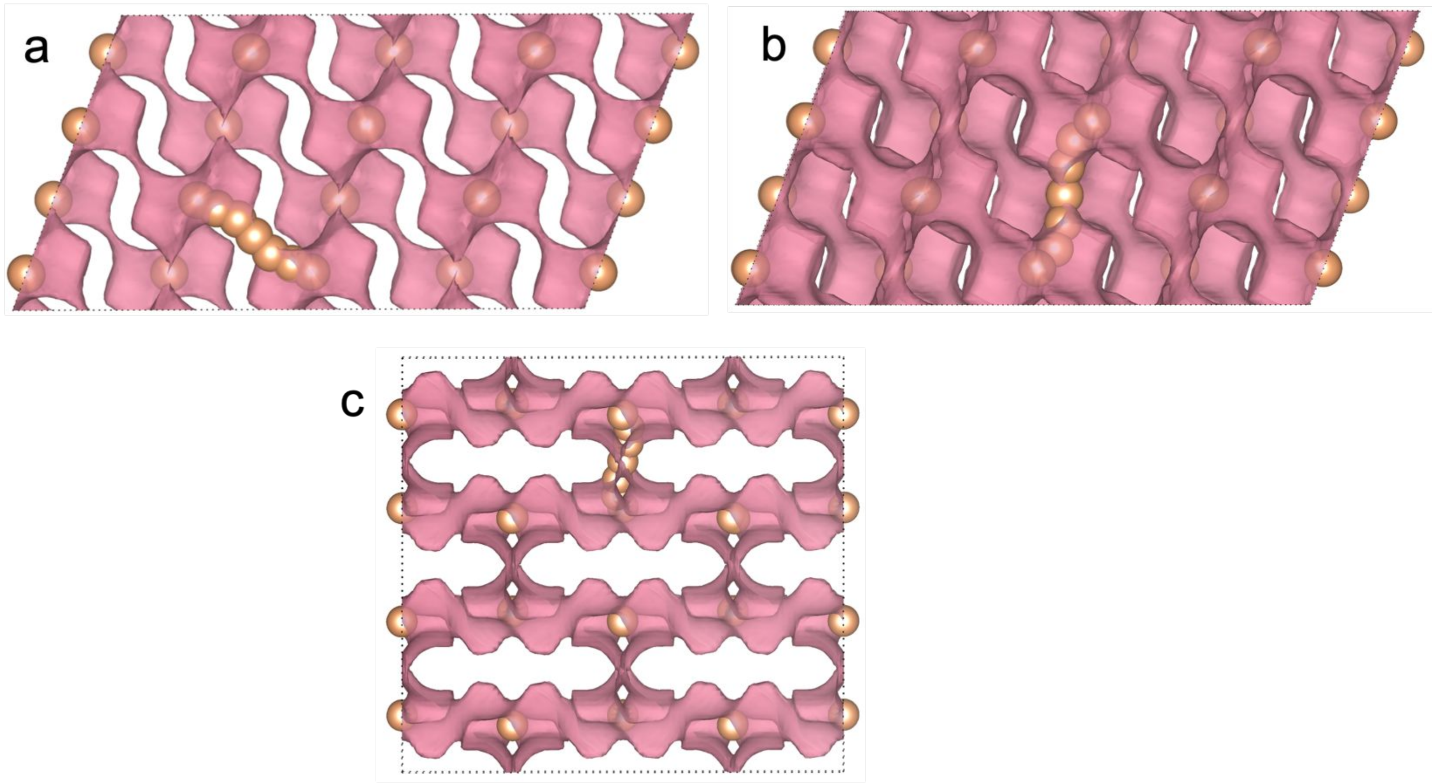

Figure SI-5: Minimum energy paths for lithium diffusion mechanism in pristine alpha spodumene from DFT-NEB superimposed on BVSE isosurface. Path 1 (a), Path 2 (b) and Path 3 (c) respectively. Color scheme: Orange (Li).

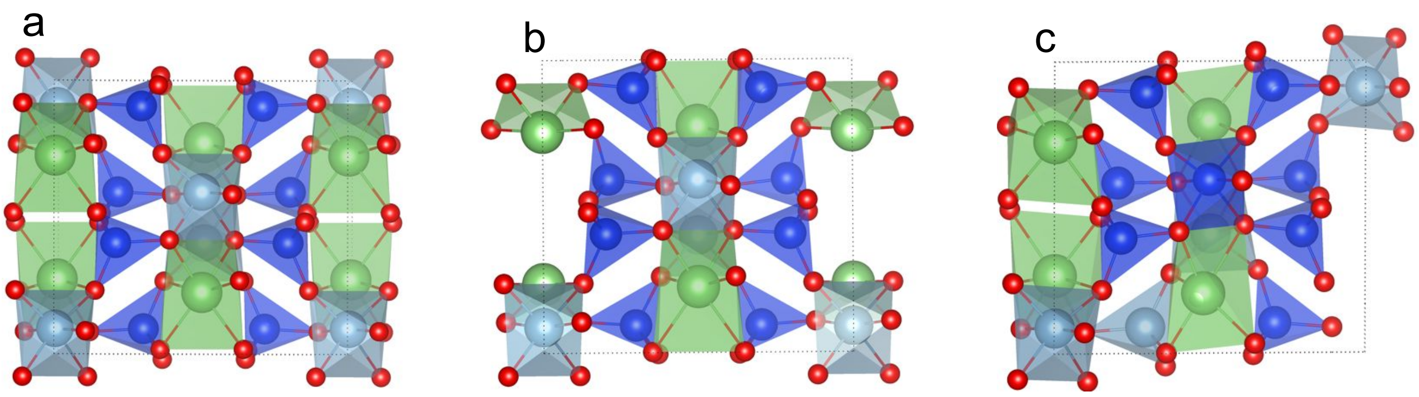

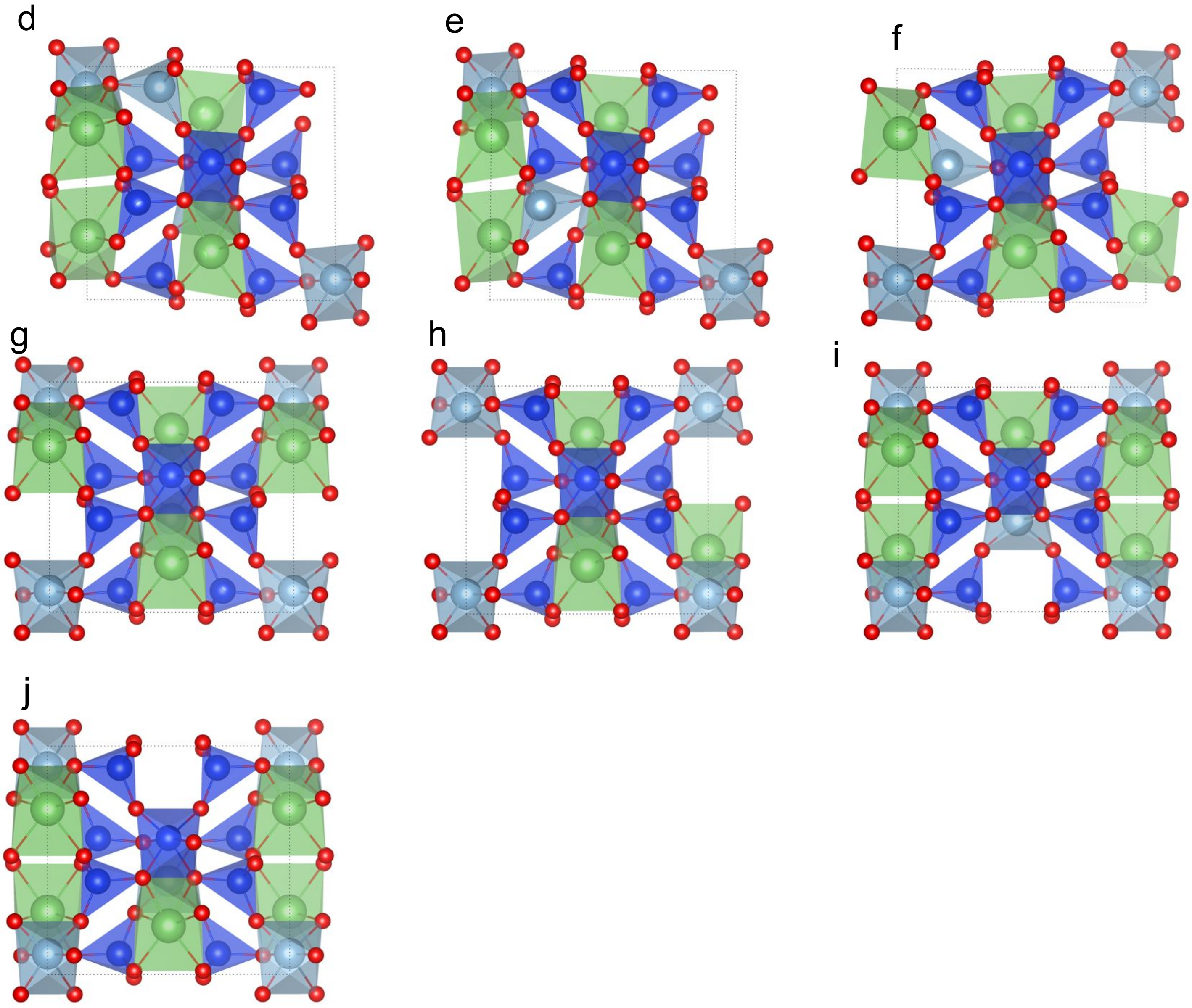

Figure SI-6: Pristine spodumene and defect spodumene structures: **(a)** pristine $LiAlSi_2O_6$, **(b)** aluminum vacancy $LiAlSi_2O_6$, **(c)-(f)** aluminum-silicon swapped $LiAlSi_2O_6$, and **(g)-(j)** aluminum and lithium substituted with silicon $LiAlSi_2O_6$. Color scheme: blue (Si), green(Li), gray(Al) and red(O).

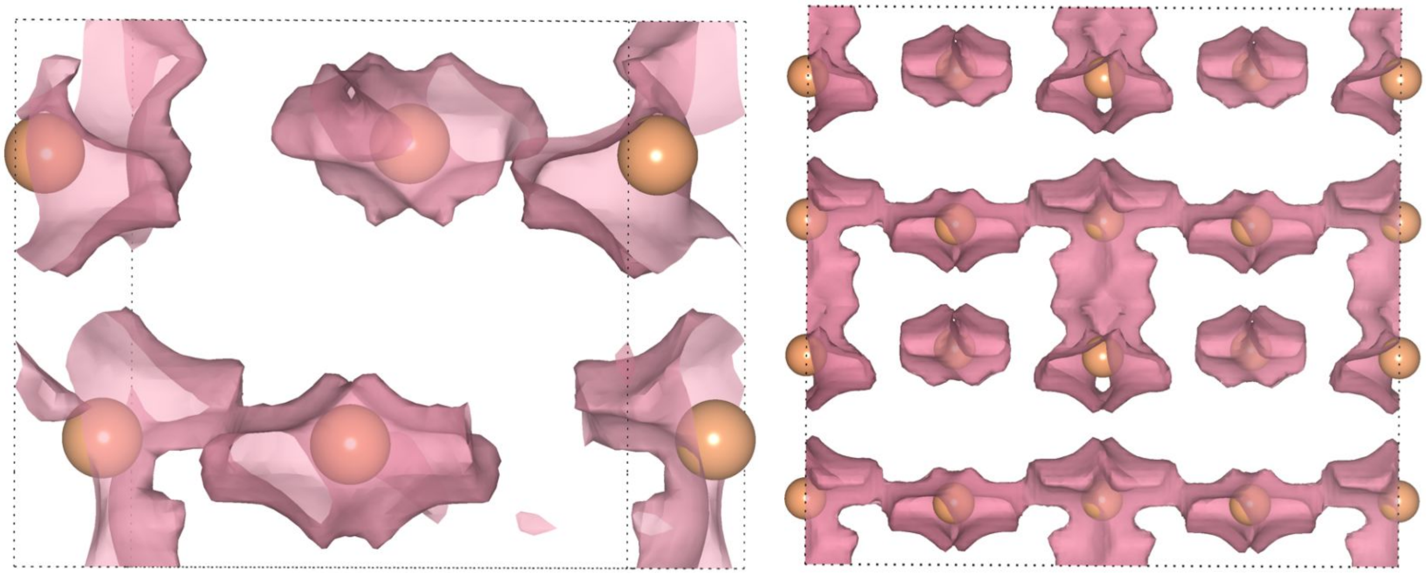

Figure SI-7(a-b): Lithium percolation barrier in alpha spodumene with aluminum vacancy (unit cell) and 2*2*2 supercell from BVSE. Color scheme: Orange (Li).

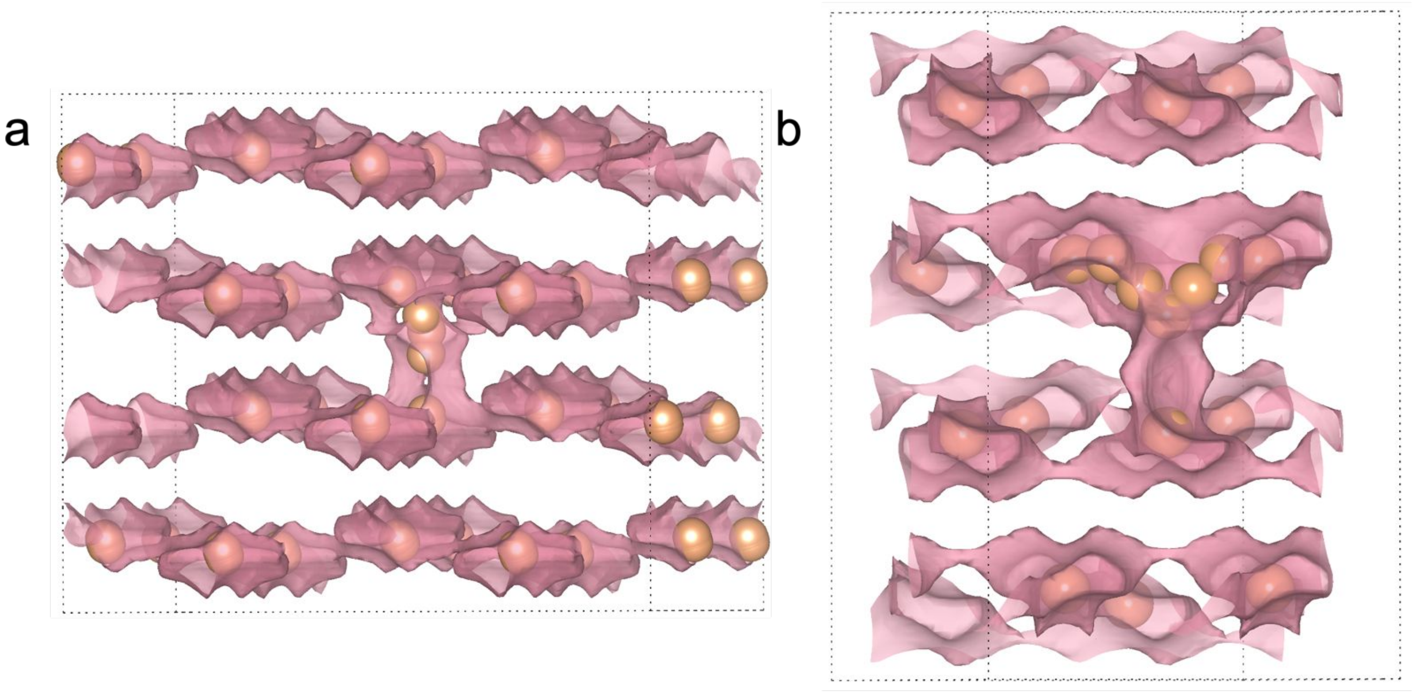

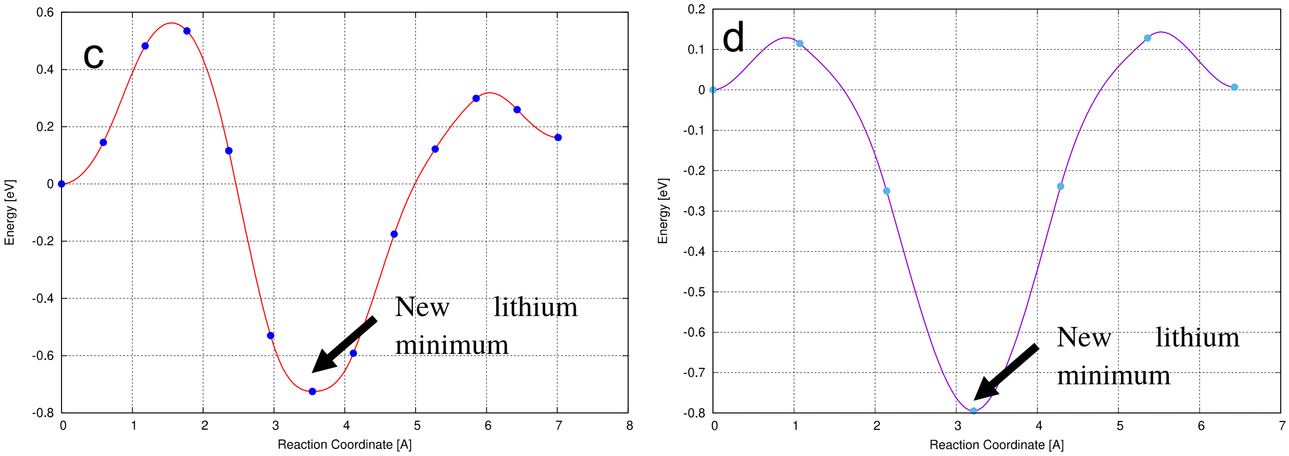

Figure SI-8: Lithium diffusion mechanism in alpha spodumene with aluminum vacancy (**a,b**) and minimum energy paths for from DFT-NEB in 2*2*2 supercell superimposed on BVSE isosurface (**c,d)**. Path 1 **(a,c)**, and Path 2 **(b,d)** respectively. Color scheme: Orange (Li).

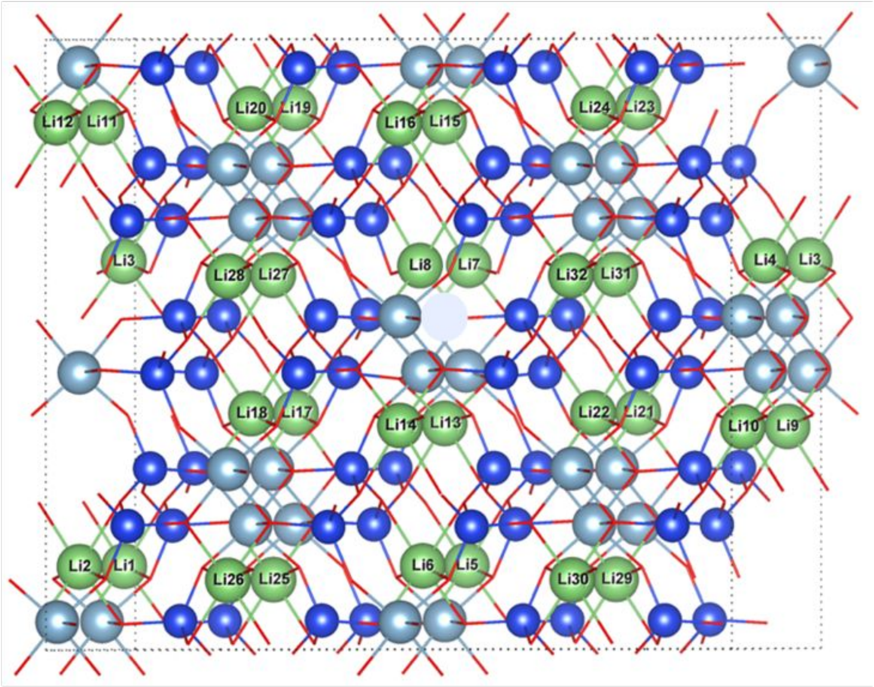

Figure SI-9: Single aluminum vacancy $LiAlSi_2O_6$ 2*2*2 supercell used for DFT-NEB simulations.

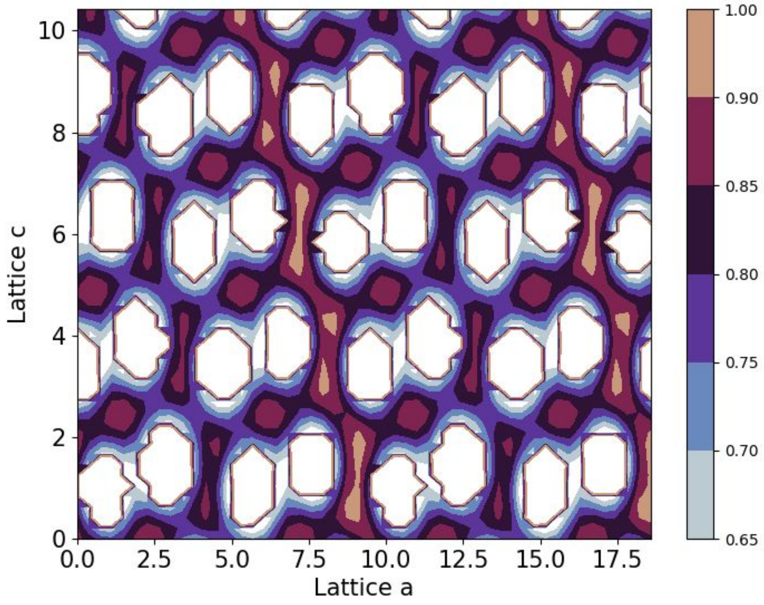

Figure SI-10: D-Map for Path 3 (see Figure 4b in main text) in $LiAlSi_2O_6$ 2*2*2 supercell perpendicular to a-c plane. D-Maps for lithium diffusion in the defect free alpha spodumene structure in the a-c plane at b (lattice height) = 2.28Å and 1.90Å are shown in Figure 5a and b = 13.55Å and 14.46Å in Figure 5a of main text.

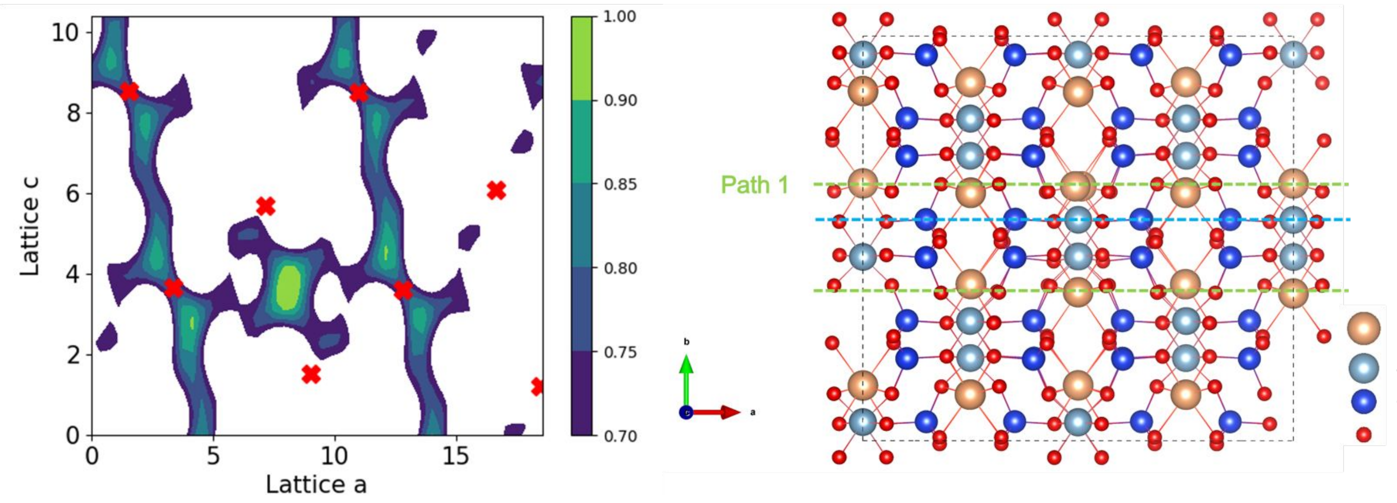

Figure SI-11: **(a)** D-Map lithium diffusion in aluminum vacancy structure, **(b)** the corresponding slice where D-Map was evaluated.

SI-1: D-Map of lithium diffusion in vacancy structure

In Figure SI-11, the diffusion of lithium happens between planes marked as Path 1 but the isosurface is demonstrated along the plane marked in blue in SI-11 (b) to show the newly created lithium site as isosurface value of 1 in D-Map in SI-10 (a). Therefore, the nearest lithium sites, in red crosses are not on the exact isosurface map.

References

(1) *https://www.iucr.org/__data/assets/file/0007/126574/bvparm2016.cif.*

(2) Brown, I. D. A Rigorous Theory of Valence. *Struct Chem* **2023**, *34* (2), 361–389. https://doi.org/10.1007/s11224-023-02128-w.

(3) Brown, I. D. Recent Developments in the Bond Valence Model of Inorganic Bonding. *Phys Chem Miner* **1987**, *15* (1), 30–34. https://doi.org/10.1007/BF00307605.

(4) Modelling the Structures of La2NiO4. **1992**, *199* (3–4), 255–272. https://doi.org/doi:10.1524/zkri.1992.199.3-4.255.

(5) *https://bvlain.readthedocs.io/en/latest/usage.html.*

(6) Momma, K.; Izumi, F. VESTA 3 for Three-Dimensional Visualization of Crystal, Volumetric and Morphology Data. *J Appl Crystallogr* **2011**, *44* (6), 1272–1276. https://doi.org/10.1107/S0021889811038970.

(7) Deng, B.; Zhong, P.; Jun, K.; Riebesell, J.; Han, K.; Bartel, C. J.; Ceder, G. CHGNet as a Pretrained Universal Neural Network Potential for Charge-Informed Atomistic Modelling. *Nat Mach Intell* **2023**, *5* (9), 1031–1041. https://doi.org/10.1038/s42256-023-00716-3.

(8) Kresse, G.; Furthmüller, J. Efficient Iterative Schemes for Ab Initio Total-Energy Calculations Using a Plane-Wave Basis Set. *Phys Rev B Condens Matter Mater Phys* **1996**, *54* (16), 11169–11186. https://doi.org/10.1103/PhysRevB.54.11169.

(9) Kresse, G.; Furthmüller, J. Efficiency of Ab-Initio Total Energy Calculations for Metals and Semiconductors Using a Plane-Wave Basis Set. *Comput Mater Sci* **1996**, *6* (1), 15–50. https://doi.org/https://doi.org/10.1016/0927-0256(96)00008-0.

(10) Sun, J.; Ruzsinszky, A.; Perdew, J. Strongly Constrained and Appropriately Normed Semilocal Density Functional. *Phys Rev Lett* **2015**, *115* (3). https://doi.org/10.1103/PhysRevLett.115.036402.

(11) Kresse, G.; Joubert, D. From Ultrasoft Pseudopotentials to the Projector Augmented-Wave Method. *Phys Rev B* **1999**, *59* (3), 1758–1775. https://doi.org/10.1103/PhysRevB.59.1758.

(12) Henkelman, G.; Jónsson, H.; Jó Nsson, H. Improved Tangent Estimate in the Nudged Elastic Band Method for Finding Minimum Energy Paths and Saddle Points. *J Chem Phys* **2000**, *113* (10), 94107–2082. https://doi.org/10.1063/1.4961868.

(13) Tang, W.; Sanville, E.; Henkelman, G. A Grid-Based Bader Analysis Algorithm without Lattice Bias. *Journal of Physics Condensed Matter* **2009**, *21* (8). https://doi.org/10.1088/0953-8984/21/8/084204.

(14) Brown, I. D. *The Chemical Bond in Inorganic Chemistry: The Bond Valence Model*; Oxford University Press, 2006. https://doi.org/10.1093/acprof:oso/9780199298815.001.0001.

(15) Moore, R. L.; Haynes, B. S.; Montoya, A. Effect of the Local Atomic Ordering on the Stability of β-Spodumene. *Inorg Chem* **2016**, *55* (13), 6426–6434. https://doi.org/10.1021/acs.inorgchem.6b00344.

(16) Schumm, R. Thermodynamic Properties of Solid Alkali Aluminosilicates at Elevated Temperatures: , National Institute of Standards and Technology, Gaithersburg, MD 1981. https://doi.org/https://doi.org/10.6028/NBS.IR.81-2343.

(17) Morgan, D.; Van der Ven, A.; Ceder, G. Li Conductivity in LixMPO4 (M = Mn, Fe, Co, Ni) Olivine Materials. *Electrochemical and Solid-State Letters* **2004**, *7* (2). https://doi.org/10.1149/1.1633511.

(18) Ouyang, C.; Shi, S.; Wang, Z.; Huang, X.; Chen, L. First-Principles Study of Li Ion Diffusion in LiFePO4. *Phys Rev B Condens Matter Mater Phys* **2004**, *69* (10). https://doi.org/10.1103/PhysRevB.69.104303.
