# Supplementary material for: Defect-Mediated Diffusion Pathways in Spodumene Accelerate Lithium Transport
Source: ACS Mater Lett. 2025 Sep 8;7(10):3388–93. doi: 10.1021/acsmaterialslett.5c00876 (PMC12505375; doi:10.1021/acsmaterialslett.5c00876)
Supplement: Supplementary file 3 [file tz5c00876_si_003.zip › 1c(t2)_sample5/1c_a.rtf]

  Table 1.  Crystal data and structure refinement for 1c_a.
Identification code 	1c_a
Empirical formula 	Al Li O6 Si2
Formula weight 	186.10
Temperature 	100(2) K
Wavelength 	0.7288 Å
Crystal system 	Monoclinic
Space group 	C2/c
Unit cell dimensions	a = 9.4556(13) Å	a= 90°.
	b = 8.3753(11) Å	b= 110.272(5)°.
	c = 5.2213(7) Å	g = 90°.
Volume	387.88(9) Å3
Z	4
Density (calculated)	3.187 Mg/m3
Absorption coefficient	1.142 mm-1
F(000)	368
Crystal size	0.250 x 0.050 x 0.010 mm3
Theta range for data collection	3.431 to 34.120°.
Index ranges	-14<=h<=14, -12<=k<=12, -7<=l<=8
Reflections collected	2441
Independent reflections	704 [R(int) = 0.0279]
Completeness to theta = 25.930°	92.9 % 
Absorption correction	Semi-empirical from equivalents
Max. and min. transmission	0.989 and 0.855
Refinement method	Full-matrix least-squares on F2
Data / restraints / parameters	704 / 0 / 47
Goodness-of-fit on F2	1.176
Final R indices [I>2sigma(I)]	R1 = 0.0385, wR2 = 0.1237
R indices (all data)	R1 = 0.0391, wR2 = 0.1244
Extinction coefficient	n/a
Largest diff. peak and hole	0.648 and -0.620 e.Å-3

 Table 2.  Atomic coordinates  ( x 104) and equivalent  isotropic displacement parameters (Å2x 103)
for 1c_a.  U(eq) is defined as one third of  the trace of the orthogonalized Uij tensor.
________________________________________________________________________________ 
	x	y	z	U(eq)
________________________________________________________________________________  
Si(1)	7060(1)	4064(1)	2431(1)	2(1)
Al(2)	5000	929(1)	2500	2(1)
O(3)	6434(1)	5141(1)	4397(2)	4(1)
O(4)	8906(1)	4175(1)	3592(2)	3(1)
O(5)	6354(1)	2325(1)	1997(2)	4(1)
Li(6)	10000	2251(4)	2500	6(1)
________________________________________________________________________________ 
 Table 3.   Bond lengths [Å] and angles [°] for  1c_a.
_____________________________________________________ 
Si(1)-O(5) 	1.5856(10)
Si(1)-O(3) 	1.6241(10)
Si(1)-O(3)#1 	1.6288(10)
Si(1)-O(4) 	1.6397(11)
Si(1)-Li(6)#2 	2.8569(15)
Si(1)-Li(6) 	3.1569(18)
Al(2)-O(5)#3 	1.8186(10)
Al(2)-O(5) 	1.8187(10)
Al(2)-O(4)#4 	1.9441(10)
Al(2)-O(4)#5 	1.9441(10)
Al(2)-O(4)#6 	1.9909(10)
Al(2)-O(4)#7 	1.9909(10)
Al(2)-Li(6)#5 	3.0228(18)
Al(2)-Li(6)#2 	3.0228(18)
Al(2)-Li(6)#6 	3.080(4)
O(3)-Li(6)#8 	2.239(3)
O(4)-Li(6) 	2.097(3)
O(5)-Li(6)#2 	2.2790(12)

O(5)-Si(1)-O(3)	111.91(5)
O(5)-Si(1)-O(3)#1	104.07(5)
O(3)-Si(1)-O(3)#1	107.38(4)
O(5)-Si(1)-O(4)	116.50(5)
O(3)-Si(1)-O(4)	108.02(5)
O(3)#1-Si(1)-O(4)	108.52(5)
O(5)-Si(1)-Li(6)#2	52.76(8)
O(3)-Si(1)-Li(6)#2	119.99(5)
O(3)#1-Si(1)-Li(6)#2	51.43(7)
O(4)-Si(1)-Li(6)#2	131.36(4)
O(5)-Si(1)-Li(6)	83.41(6)
O(3)-Si(1)-Li(6)	140.54(4)
O(3)#1-Si(1)-Li(6)	103.25(4)
O(4)-Si(1)-Li(6)	37.11(6)
Li(6)#2-Si(1)-Li(6)	98.481(17)
O(5)#3-Al(2)-O(5)	100.00(6)
O(5)#3-Al(2)-O(4)#4	92.02(4)
O(5)-Al(2)-O(4)#4	91.28(4)
O(5)#3-Al(2)-O(4)#5	91.28(4)
O(5)-Al(2)-O(4)#5	92.02(4)
O(4)#4-Al(2)-O(4)#5	174.87(6)
O(5)#3-Al(2)-O(4)#6	88.36(4)
O(5)-Al(2)-O(4)#6	167.82(5)
O(4)#4-Al(2)-O(4)#6	97.30(4)
O(4)#5-Al(2)-O(4)#6	78.86(5)
O(5)#3-Al(2)-O(4)#7	167.82(5)
O(5)-Al(2)-O(4)#7	88.36(4)
O(4)#4-Al(2)-O(4)#7	78.86(5)
O(4)#5-Al(2)-O(4)#7	97.30(4)
O(4)#6-Al(2)-O(4)#7	84.85(6)
O(5)#3-Al(2)-Li(6)#5	48.74(4)
O(5)-Al(2)-Li(6)#5	90.66(5)
O(4)#4-Al(2)-Li(6)#5	140.34(4)
O(4)#5-Al(2)-Li(6)#5	43.55(5)
O(4)#6-Al(2)-Li(6)#5	88.24(6)
O(4)#7-Al(2)-Li(6)#5	140.79(5)
O(5)#3-Al(2)-Li(6)#2	90.65(5)
O(5)-Al(2)-Li(6)#2	48.74(4)
O(4)#4-Al(2)-Li(6)#2	43.55(5)
O(4)#5-Al(2)-Li(6)#2	140.34(4)
O(4)#6-Al(2)-Li(6)#2	140.79(5)
O(4)#7-Al(2)-Li(6)#2	88.24(6)
Li(6)#5-Al(2)-Li(6)#2	119.46(12)
O(5)#3-Al(2)-Li(6)#6	130.00(3)
O(5)-Al(2)-Li(6)#6	130.00(3)
O(4)#4-Al(2)-Li(6)#6	87.43(3)
O(4)#5-Al(2)-Li(6)#6	87.43(3)
O(4)#6-Al(2)-Li(6)#6	42.43(3)
O(4)#7-Al(2)-Li(6)#6	42.43(3)
Li(6)#5-Al(2)-Li(6)#6	120.27(6)
Li(6)#2-Al(2)-Li(6)#6	120.27(6)
Si(1)-O(3)-Si(1)#9	138.81(7)
Si(1)-O(3)-Li(6)#8	117.15(6)
Si(1)#9-O(3)-Li(6)#8	93.90(6)
Si(1)-O(4)-Al(2)#5	119.77(6)
Si(1)-O(4)-Al(2)#10	121.87(5)
Al(2)#5-O(4)-Al(2)#10	101.14(5)
Si(1)-O(4)-Li(6)	114.74(7)
Al(2)#5-O(4)-Li(6)	96.75(5)
Al(2)#10-O(4)-Li(6)	97.75(8)
Si(1)-O(5)-Al(2)	148.48(6)
Si(1)-O(5)-Li(6)#2	93.60(10)
Al(2)-O(5)-Li(6)#2	94.40(7)
O(4)-Li(6)-O(4)#11	79.65(14)
O(4)-Li(6)-O(3)#7	116.50(4)
O(4)#11-Li(6)-O(3)#7	140.02(4)
O(4)-Li(6)-O(3)#12	140.02(4)
O(4)#11-Li(6)-O(3)#12	116.50(4)
O(3)#7-Li(6)-O(3)#12	75.76(13)
O(4)-Li(6)-O(5)#13	75.78(8)
O(4)#11-Li(6)-O(5)#13	90.37(9)
O(3)#7-Li(6)-O(5)#13	128.06(12)
O(3)#12-Li(6)-O(5)#13	68.23(5)
O(4)-Li(6)-O(5)#2	90.37(9)
O(4)#11-Li(6)-O(5)#2	75.78(8)
O(3)#7-Li(6)-O(5)#2	68.23(5)
O(3)#12-Li(6)-O(5)#2	128.06(12)
O(5)#13-Li(6)-O(5)#2	162.09(19)
O(4)-Li(6)-Si(1)#2	107.13(5)
O(4)#11-Li(6)-Si(1)#2	107.33(5)
O(3)#7-Li(6)-Si(1)#2	34.67(3)
O(3)#12-Li(6)-Si(1)#2	102.33(12)
O(5)#13-Li(6)-Si(1)#2	162.30(13)
O(5)#2-Li(6)-Si(1)#2	33.63(3)
O(4)-Li(6)-Si(1)#13	107.33(5)
O(4)#11-Li(6)-Si(1)#13	107.13(5)
O(3)#7-Li(6)-Si(1)#13	102.33(12)
O(3)#12-Li(6)-Si(1)#13	34.67(3)
O(5)#13-Li(6)-Si(1)#13	33.63(3)
O(5)#2-Li(6)-Si(1)#13	162.30(13)
Si(1)#2-Li(6)-Si(1)#13	134.63(14)
O(4)-Li(6)-Al(2)#5	39.69(4)
O(4)#11-Li(6)-Al(2)#5	89.72(11)
O(3)#7-Li(6)-Al(2)#5	126.78(4)
O(3)#12-Li(6)-Al(2)#5	101.37(3)
O(5)#13-Li(6)-Al(2)#5	36.86(4)
O(5)#2-Li(6)-Al(2)#5	130.03(12)
Si(1)#2-Li(6)-Al(2)#5	140.24(3)
Si(1)#13-Li(6)-Al(2)#5	67.671(17)
O(4)-Li(6)-Al(2)#2	89.72(11)
O(4)#11-Li(6)-Al(2)#2	39.69(4)
O(3)#7-Li(6)-Al(2)#2	101.37(3)
O(3)#12-Li(6)-Al(2)#2	126.78(4)
O(5)#13-Li(6)-Al(2)#2	130.03(12)
O(5)#2-Li(6)-Al(2)#2	36.86(4)
Si(1)#2-Li(6)-Al(2)#2	67.671(16)
Si(1)#13-Li(6)-Al(2)#2	140.24(3)
Al(2)#5-Li(6)-Al(2)#2	119.46(12)
O(4)-Li(6)-Al(2)#10	39.82(7)
O(4)#11-Li(6)-Al(2)#10	39.82(7)
O(3)#7-Li(6)-Al(2)#10	142.12(6)
O(3)#12-Li(6)-Al(2)#10	142.12(6)
O(5)#13-Li(6)-Al(2)#10	81.05(9)
O(5)#2-Li(6)-Al(2)#10	81.05(9)
Si(1)#2-Li(6)-Al(2)#10	112.69(7)
Si(1)#13-Li(6)-Al(2)#10	112.69(7)
Al(2)#5-Li(6)-Al(2)#10	59.73(6)
Al(2)#2-Li(6)-Al(2)#10	59.73(6)
O(4)-Li(6)-Si(1)	28.15(3)
O(4)#11-Li(6)-Si(1)	98.22(12)
O(3)#7-Li(6)-Si(1)	88.77(3)
O(3)#12-Li(6)-Si(1)	141.33(7)
O(5)#13-Li(6)-Si(1)	96.60(7)
O(5)#2-Li(6)-Si(1)	74.65(5)
Si(1)#2-Li(6)-Si(1)	81.520(17)
Si(1)#13-Li(6)-Si(1)	121.23(2)
Al(2)#5-Li(6)-Si(1)	60.26(4)
Al(2)#2-Li(6)-Si(1)	90.64(7)
Al(2)#10-Li(6)-Si(1)	61.25(6)
_____________________________________________________________ 
Symmetry transformations used to generate equivalent atoms: 
#1 x,-y+1,z-1/2    #2 -x+3/2,-y+1/2,-z    #3 -x+1,y,-z+1/2      
#4 x-1/2,-y+1/2,z-1/2    #5 -x+3/2,-y+1/2,-z+1      
#6 x-1/2,y-1/2,z    #7 -x+3/2,y-1/2,-z+1/2    #8 x-1/2,y+1/2,z      
#9 x,-y+1,z+1/2    #10 x+1/2,y+1/2,z    #11 -x+2,y,-z+1/2      
#12 x+1/2,y-1/2,z    #13 x+1/2,-y+1/2,z+1/2      

 Table 4.   Anisotropic displacement parameters  (Å2x 103) for 1c_a.  The anisotropic
displacement factor exponent takes the form:  -2p2[ h2 a*2U11 + ...  + 2 h k a* b* U12 ]
______________________________________________________________________________ 
	U11	U22 	U33	U23	U13	U12
______________________________________________________________________________ 
Si(1)	2(1) 	2(1)	2(1) 	0(1)	1(1) 	0(1)
Al(2)	2(1) 	2(1)	3(1) 	0	1(1) 	0
O(3)	4(1) 	6(1)	3(1) 	-2(1)	2(1) 	0(1)
O(4)	3(1) 	3(1)	4(1) 	0(1)	1(1) 	0(1)
O(5)	5(1) 	2(1)	6(1) 	0(1)	3(1) 	-1(1)
Li(6)	4(1) 	7(1)	6(2) 	0	2(1) 	0
______________________________________________________________________________ 
 
 
