# Supplementary material for: Defect-Mediated Diffusion Pathways in Spodumene Accelerate Lithium Transport
Source: ACS Mater Lett. 2025 Sep 8;7(10):3388–93. doi: 10.1021/acsmaterialslett.5c00876 (PMC12505375; doi:10.1021/acsmaterialslett.5c00876)
Supplement: Supplementary file 3 [file tz5c00876_si_003.zip › 1d_sample 1/1d_a.rtf]

  Table 1.  Crystal data and structure refinement for 1d_a.
Identification code 	1d_a
Empirical formula 	Al Li O6 Si2
Formula weight 	186.10
Temperature 	100(2) K
Wavelength 	0.7288 Å
Crystal system 	Monoclinic
Space group 	C2/c
Unit cell dimensions	a = 9.4702(10) Å	a= 90°.
	b = 8.3885(8) Å	b= 110.281(4)°.
	c = 5.2295(5) Å	g = 90°.
Volume	389.68(7) Å3
Z	4
Density (calculated)	3.172 Mg/m3
Absorption coefficient	1.137 mm-1
F(000)	368
Crystal size	0.180 x 0.030 x 0.030 mm3
Theta range for data collection	3.425 to 36.474°.
Index ranges	-15<=h<=15, -13<=k<=13, -8<=l<=7
Reflections collected	3710
Independent reflections	849 [R(int) = 0.0378]
Completeness to theta = 25.930°	97.5 % 
Absorption correction	Semi-empirical from equivalents
Max. and min. transmission	0.967 and 0.855
Refinement method	Full-matrix least-squares on F2
Data / restraints / parameters	849 / 0 / 47
Goodness-of-fit on F2	1.139
Final R indices [I>2sigma(I)]	R1 = 0.0358, wR2 = 0.1144
R indices (all data)	R1 = 0.0373, wR2 = 0.1162
Extinction coefficient	n/a
Largest diff. peak and hole	0.867 and -0.756 e.Å-3

 Table 2.  Atomic coordinates  ( x 104) and equivalent  isotropic displacement parameters (Å2x 103)
for 1d_a.  U(eq) is defined as one third of  the trace of the orthogonalized Uij tensor.
________________________________________________________________________________ 
	x	y	z	U(eq)
________________________________________________________________________________  
Si(1)	7060(1)	4065(1)	2431(1)	2(1)
Al(2)	5000	931(1)	2500	1(1)
O(3)	6434(1)	5138(1)	4398(2)	4(1)
O(4)	8903(1)	4174(1)	3588(2)	3(1)
O(5)	6353(1)	2326(1)	1993(2)	4(1)
Li(6)	10000	2255(4)	2500	4(1)
________________________________________________________________________________ 
 Table 3.   Bond lengths [Å] and angles [°] for  1d_a.
_____________________________________________________ 
Si(1)-O(5) 	1.5878(9)
Si(1)-O(3) 	1.6257(9)
Si(1)-O(3)#1 	1.6314(10)
Si(1)-O(4) 	1.6397(10)
Si(1)-Li(6)#2 	2.8621(13)
Si(1)-Li(6) 	3.1609(16)
Al(2)-O(5) 	1.8215(9)
Al(2)-O(5)#3 	1.8215(9)
Al(2)-O(4)#4 	1.9492(9)
Al(2)-O(4)#5 	1.9492(9)
Al(2)-O(4)#6 	1.9960(9)
Al(2)-O(4)#7 	1.9960(9)
Al(2)-Li(6)#5 	3.0257(16)
Al(2)-Li(6)#2 	3.0257(16)
Al(2)-Li(6)#6 	3.084(3)
O(3)-Li(6)#8 	2.247(3)
O(4)-Li(6) 	2.099(3)
O(5)-Li(6)#2 	2.2802(11)

O(5)-Si(1)-O(3)	111.82(5)
O(5)-Si(1)-O(3)#1	104.09(5)
O(3)-Si(1)-O(3)#1	107.40(3)
O(5)-Si(1)-O(4)	116.51(5)
O(3)-Si(1)-O(4)	108.08(5)
O(3)#1-Si(1)-O(4)	108.50(5)
O(5)-Si(1)-Li(6)#2	52.65(7)
O(3)-Si(1)-Li(6)#2	120.02(5)
O(3)#1-Si(1)-Li(6)#2	51.56(6)
O(4)-Si(1)-Li(6)#2	131.29(4)
O(5)-Si(1)-Li(6)	83.47(6)
O(3)-Si(1)-Li(6)	140.51(4)
O(3)#1-Si(1)-Li(6)	103.28(4)
O(4)-Si(1)-Li(6)	37.05(5)
Li(6)#2-Si(1)-Li(6)	98.459(15)
O(5)-Al(2)-O(5)#3	99.99(6)
O(5)-Al(2)-O(4)#4	91.27(4)
O(5)#3-Al(2)-O(4)#4	92.05(4)
O(5)-Al(2)-O(4)#5	92.05(4)
O(5)#3-Al(2)-O(4)#5	91.27(4)
O(4)#4-Al(2)-O(4)#5	174.83(6)
O(5)-Al(2)-O(4)#6	167.93(4)
O(5)#3-Al(2)-O(4)#6	88.35(4)
O(4)#4-Al(2)-O(4)#6	97.18(4)
O(4)#5-Al(2)-O(4)#6	78.95(4)
O(5)-Al(2)-O(4)#7	88.35(4)
O(5)#3-Al(2)-O(4)#7	167.93(4)
O(4)#4-Al(2)-O(4)#7	78.95(4)
O(4)#5-Al(2)-O(4)#7	97.18(4)
O(4)#6-Al(2)-O(4)#7	84.85(5)
O(5)-Al(2)-Li(6)#5	90.74(5)
O(5)#3-Al(2)-Li(6)#5	48.71(4)
O(4)#4-Al(2)-Li(6)#5	140.36(4)
O(4)#5-Al(2)-Li(6)#5	43.56(5)
O(4)#6-Al(2)-Li(6)#5	88.23(5)
O(4)#7-Al(2)-Li(6)#5	140.68(4)
O(5)-Al(2)-Li(6)#2	48.71(4)
O(5)#3-Al(2)-Li(6)#2	90.74(5)
O(4)#4-Al(2)-Li(6)#2	43.56(5)
O(4)#5-Al(2)-Li(6)#2	140.36(4)
O(4)#6-Al(2)-Li(6)#2	140.68(4)
O(4)#7-Al(2)-Li(6)#2	88.23(5)
Li(6)#5-Al(2)-Li(6)#2	119.58(10)
O(5)-Al(2)-Li(6)#6	130.01(3)
O(5)#3-Al(2)-Li(6)#6	130.01(3)
O(4)#4-Al(2)-Li(6)#6	87.42(3)
O(4)#5-Al(2)-Li(6)#6	87.42(3)
O(4)#6-Al(2)-Li(6)#6	42.43(3)
O(4)#7-Al(2)-Li(6)#6	42.43(3)
Li(6)#5-Al(2)-Li(6)#6	120.21(5)
Li(6)#2-Al(2)-Li(6)#6	120.21(5)
Si(1)-O(3)-Si(1)#9	138.84(6)
Si(1)-O(3)-Li(6)#8	117.07(5)
Si(1)#9-O(3)-Li(6)#8	93.78(6)
Si(1)-O(4)-Al(2)#5	119.79(5)
Si(1)-O(4)-Al(2)#10	121.93(5)
Al(2)#5-O(4)-Al(2)#10	101.05(4)
Si(1)-O(4)-Li(6)	114.88(6)
Al(2)#5-O(4)-Li(6)	96.66(4)
Al(2)#10-O(4)-Li(6)	97.67(7)
Si(1)-O(5)-Al(2)	148.45(6)
Si(1)-O(5)-Li(6)#2	93.73(9)
Al(2)-O(5)-Li(6)#2	94.40(6)
O(4)-Li(6)-O(4)#11	79.81(12)
O(4)-Li(6)-O(3)#7	116.47(3)
O(4)#11-Li(6)-O(3)#7	140.06(4)
O(4)-Li(6)-O(3)#12	140.06(4)
O(4)#11-Li(6)-O(3)#12	116.47(3)
O(3)#7-Li(6)-O(3)#12	75.58(11)
O(4)-Li(6)-O(5)#13	75.91(7)
O(4)#11-Li(6)-O(5)#13	90.40(8)
O(3)#7-Li(6)-O(5)#13	127.94(11)
O(3)#12-Li(6)-O(5)#13	68.21(5)
O(4)-Li(6)-O(5)#2	90.40(8)
O(4)#11-Li(6)-O(5)#2	75.91(7)
O(3)#7-Li(6)-O(5)#2	68.21(5)
O(3)#12-Li(6)-O(5)#2	127.94(11)
O(5)#13-Li(6)-O(5)#2	162.27(16)
O(4)-Li(6)-Si(1)#2	107.10(4)
O(4)#11-Li(6)-Si(1)#2	107.42(4)
O(3)#7-Li(6)-Si(1)#2	34.66(3)
O(3)#12-Li(6)-Si(1)#2	102.20(11)
O(5)#13-Li(6)-Si(1)#2	162.18(11)
O(5)#2-Li(6)-Si(1)#2	33.61(3)
O(4)-Li(6)-Si(1)#13	107.42(4)
O(4)#11-Li(6)-Si(1)#13	107.10(4)
O(3)#7-Li(6)-Si(1)#13	102.20(11)
O(3)#12-Li(6)-Si(1)#13	34.66(3)
O(5)#13-Li(6)-Si(1)#13	33.61(3)
O(5)#2-Li(6)-Si(1)#13	162.18(11)
Si(1)#2-Li(6)-Si(1)#13	134.50(12)
O(4)-Li(6)-Al(2)#5	39.78(3)
O(4)#11-Li(6)-Al(2)#5	89.80(9)
O(3)#7-Li(6)-Al(2)#5	126.76(4)
O(3)#12-Li(6)-Al(2)#5	101.35(3)
O(5)#13-Li(6)-Al(2)#5	36.89(4)
O(5)#2-Li(6)-Al(2)#5	130.14(10)
Si(1)#2-Li(6)-Al(2)#5	140.26(2)
Si(1)#13-Li(6)-Al(2)#5	67.678(14)
O(4)-Li(6)-Al(2)#2	89.80(9)
O(4)#11-Li(6)-Al(2)#2	39.78(3)
O(3)#7-Li(6)-Al(2)#2	101.35(3)
O(3)#12-Li(6)-Al(2)#2	126.76(4)
O(5)#13-Li(6)-Al(2)#2	130.14(10)
O(5)#2-Li(6)-Al(2)#2	36.89(4)
Si(1)#2-Li(6)-Al(2)#2	67.678(14)
Si(1)#13-Li(6)-Al(2)#2	140.26(2)
Al(2)#5-Li(6)-Al(2)#2	119.58(10)
O(4)-Li(6)-Al(2)#10	39.90(6)
O(4)#11-Li(6)-Al(2)#10	39.90(6)
O(3)#7-Li(6)-Al(2)#10	142.21(5)
O(3)#12-Li(6)-Al(2)#10	142.21(5)
O(5)#13-Li(6)-Al(2)#10	81.13(8)
O(5)#2-Li(6)-Al(2)#10	81.13(8)
Si(1)#2-Li(6)-Al(2)#10	112.75(6)
Si(1)#13-Li(6)-Al(2)#10	112.75(6)
Al(2)#5-Li(6)-Al(2)#10	59.79(5)
Al(2)#2-Li(6)-Al(2)#10	59.79(5)
O(4)-Li(6)-Si(1)#11	98.35(11)
O(4)#11-Li(6)-Si(1)#11	28.07(3)
O(3)#7-Li(6)-Si(1)#11	141.25(6)
O(3)#12-Li(6)-Si(1)#11	88.82(2)
O(5)#13-Li(6)-Si(1)#11	74.70(4)
O(5)#2-Li(6)-Si(1)#11	96.65(6)
Si(1)#2-Li(6)-Si(1)#11	121.245(17)
Si(1)#13-Li(6)-Si(1)#11	81.541(14)
Al(2)#5-Li(6)-Si(1)#11	90.70(6)
Al(2)#2-Li(6)-Si(1)#11	60.28(3)
Al(2)#10-Li(6)-Si(1)#11	61.28(5)
_____________________________________________________________ 
Symmetry transformations used to generate equivalent atoms: 
#1 x,-y+1,z-1/2    #2 -x+3/2,-y+1/2,-z    #3 -x+1,y,-z+1/2      
#4 x-1/2,-y+1/2,z-1/2    #5 -x+3/2,-y+1/2,-z+1      
#6 x-1/2,y-1/2,z    #7 -x+3/2,y-1/2,-z+1/2    #8 x-1/2,y+1/2,z      
#9 x,-y+1,z+1/2    #10 x+1/2,y+1/2,z    #11 -x+2,y,-z+1/2      
#12 x+1/2,y-1/2,z    #13 x+1/2,-y+1/2,z+1/2      

 Table 4.   Anisotropic displacement parameters  (Å2x 103) for 1d_a.  The anisotropic
displacement factor exponent takes the form:  -2p2[ h2 a*2U11 + ...  + 2 h k a* b* U12 ]
______________________________________________________________________________ 
	U11	U22 	U33	U23	U13	U12
______________________________________________________________________________ 
Si(1)	2(1) 	2(1)	2(1) 	0(1)	1(1) 	0(1)
Al(2)	1(1) 	1(1)	2(1) 	0	1(1) 	0
O(3)	3(1) 	6(1)	2(1) 	-2(1)	1(1) 	0(1)
O(4)	2(1) 	4(1)	3(1) 	0(1)	0(1) 	0(1)
O(5)	4(1) 	2(1)	5(1) 	0(1)	2(1) 	-2(1)
Li(6)	4(1) 	4(1)	5(1) 	0	1(1) 	0
______________________________________________________________________________ 
 
 
