# Supplementary material for: Defect-Mediated Diffusion Pathways in Spodumene Accelerate Lithium Transport
Source: ACS Mater Lett. 2025 Sep 8;7(10):3388–93. doi: 10.1021/acsmaterialslett.5c00876 (PMC12505375; doi:10.1021/acsmaterialslett.5c00876)

# checkCIF/PLATON report

Structure factors have been supplied for datablock(s) 1e_a

THIS REPORT IS FOR GUIDANCE ONLY. IF USED AS PART OF A REVIEW PROCEDURE FOR PUBLICATION, IT SHOULD NOT REPLACE THE EXPERTISE OF AN EXPERIENCED CRYSTALLOGRAPHIC REFEREE.

No syntax errors found. CIF dictionary Interpreting this report

# Datablock: 1e_a

---

| | | | |
|---|---|---|---|
| Bond precision: | Si- O = 0.0006 A | | Wavelength=0.72880 |
| Cell: | a=9.4667(9) | b=8.3854(8) | c=5.2222(5) |
| | alpha=90 | beta=110.211(3) | gamma=90 |
| Temperature: | 100 K | | |

| | Calculated | Reported |
|---|---|---|
| Volume | 389.02(6) | 389.02(6) |
| Space group | C 2/c | C 2/c |
| Hall group | -C 2yc | -C 2yc |
| Moiety formula | Al2 O12 Si4, 2(Li) | ? |
| Sum formula | Al2 Li2 O12 Si4 | Al2 Li2 O12 Si4 |
| Mr | 372.20 | 372.20 |
| Dx,g cm-3 | 3.178 | 3.177 |
| Z | 2 | 2 |
| Mu (mm-1) | 1.139 | 1.139 |
| F000 | 368.0 | 368.0 |
| F000’ | 369.18 | |
| h,k,lmax | 15,13,8 | 15,13,8 |
| Nref | 896 | 863 |
| Tmin,Tmax | 0.934,0.955 | 0.899,0.956 |
| Tmin’ | 0.833 | |

Correction method= # Reported T Limits: Tmin=0.899 Tmax=0.956
AbsCorr = MULTI-SCAN

Data completeness= 0.963 Theta(max)= 36.572

R(reflections)= 0.0199( 825) wR2(reflections)= 0.0627( 863)

S = 1.143 Npar= 47

The following ALERTS were generated. Each ALERT has the format
**test-name_ALERT_alert-type_alert-level.**
Click on the hyperlinks for more details of the test.

## Alert level C

```
PLAT911_ALERT_3_C Missing FCF Refl Between Thmin & STh/L=    0.600          6 Report
                0  2  0,   0  6  0,  -2  2  1,   1  3  1,  -3  1  2,   0  0  2,
```

## Alert level G

```
ABSMU01_ALERT_1_G  Calculation of _exptl_absorpt_correction_mu
                not performed for this radiation type.
PLAT004_ALERT_5_G Polymeric Structure Found with Maximum Dimension          3 Info
PLAT128_ALERT_4_G Alternate Setting for Input Space Group     C2/c       I2/a Note
PLAT396_ALERT_2_G Deviating Si-O-Si  Angle From 150 for O3       .      138.7 Degree
PLAT794_ALERT_5_G Tentative Bond Valency for Al2       (III)     .       2.72 Info
PLAT912_ALERT_4_G Missing # of FCF Reflections Above STh/L=  0.600         27 Note
PLAT913_ALERT_3_G Missing # of Very Strong Reflections in FCF ....          2 Note
               -2  2  1,   0  0  2,
PLAT933_ALERT_2_G Number of HKL-OMIT Records in Embedded .res File          2 Note
                1  3  1,  -3  1  2,
PLAT969_ALERT_5_G The 'Henn et al.' R-Factor-gap value ..........      4.066 Note
              Predicted wR2: Based on SigI**2  1.54 or SHELX Weight  5.48
```

0 **ALERT level A** = Most likely a serious problem - resolve or explain
0 **ALERT level B** = A potentially serious problem, consider carefully
1 **ALERT level C** = Check. Ensure it is not caused by an omission or oversight
9 **ALERT level G** = General information/check it is not something unexpected

1 ALERT type 1 CIF construction/syntax error, inconsistent or missing data
2 ALERT type 2 Indicator that the structure model may be wrong or deficient
2 ALERT type 3 Indicator that the structure quality may be low
2 ALERT type 4 Improvement, methodology, query or suggestion
3 ALERT type 5 Informative message, check

It is advisable to attempt to resolve as many as possible of the alerts in all categories. Often the minor alerts point to easily fixed oversights, errors and omissions in your CIF or refinement strategy, so attention to these fine details can be worthwhile. In order to resolve some of the more serious problems it may be necessary to carry out additional measurements or structure refinements. However, the purpose of your study may justify the reported deviations and the more serious of these should normally be commented upon in the discussion or experimental section of a paper or in the "special_details" fields of the CIF. checkCIF was carefully designed to identify outliers and unusual parameters, but every test has its limitations and alerts that are not important in a particular case may appear. Conversely, the absence of alerts does not guarantee there are no aspects of the results needing attention. It is up to the individual to critically assess their own results and, if necessary, seek expert advice.

**Publication of your CIF in IUCr journals**

A basic structural check has been run on your CIF. These basic checks will be run on all CIFs submitted for publication in IUCr journals (*Acta Crystallographica*, *Journal of Applied Crystallography*, *Journal of Synchrotron Radiation*); however, if you intend to submit to *Acta Crystallographica Section C* or *E* or *IUCrData*, you should make sure that full publication checks are run on the final version of your CIF prior to submission.

**Publication of your CIF in other journals**

Please refer to the *Notes for Authors* of the relevant journal for any special instructions relating to CIF submission.

---

**PLATON version of 02/02/2025; check.def file version of 02/02/2025**

**Datablock 1e_a** - ellipsoid plot

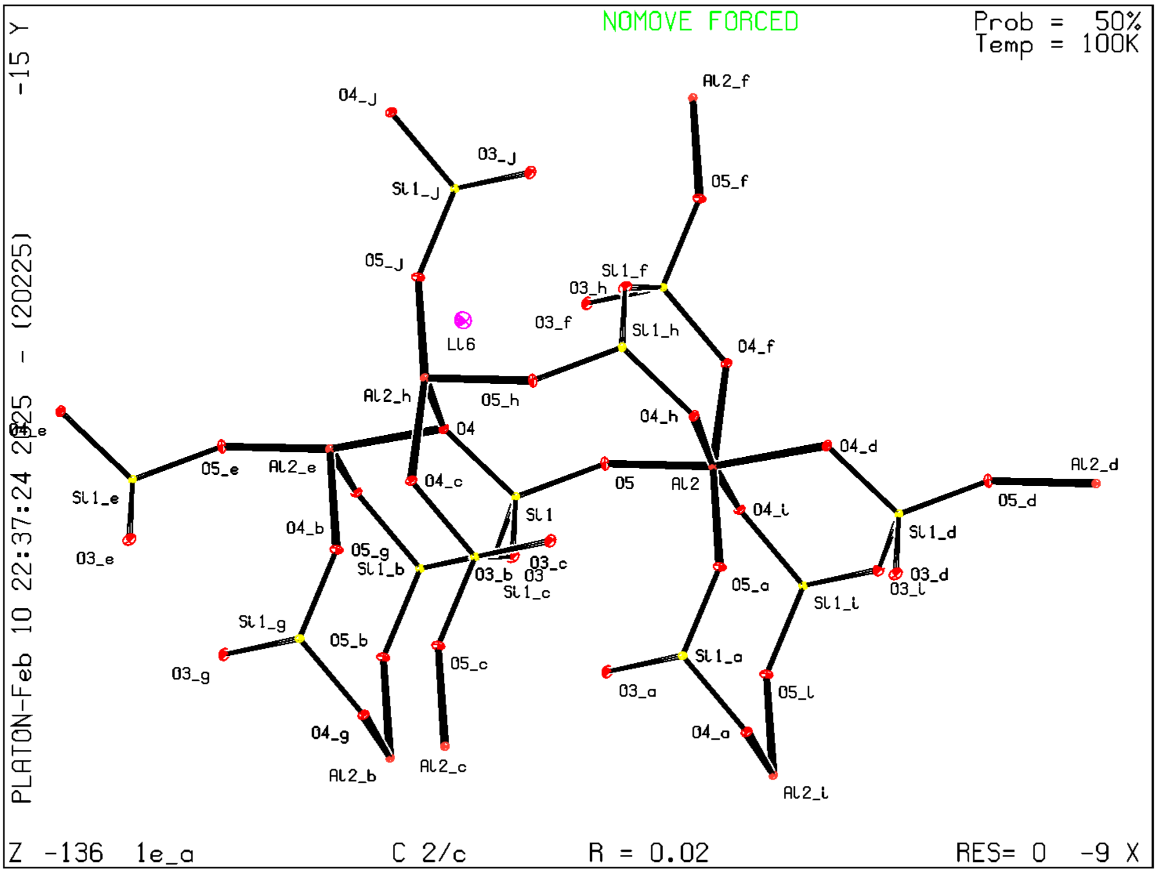

Supplement: Supplementary file 3 [file tz5c00876_si_003.zip › 1e_sample2/checkcif.pdf]
