# Supplementary material for: Defect-Mediated Diffusion Pathways in Spodumene Accelerate Lithium Transport
Source: ACS Mater Lett. 2025 Sep 8;7(10):3388–93. doi: 10.1021/acsmaterialslett.5c00876 (PMC12505375; doi:10.1021/acsmaterialslett.5c00876)
Supplement: Supplementary file 3 [file tz5c00876_si_003.zip › 1e_sample2/1e_a.rtf]

  Table 1.  Crystal data and structure refinement for 1e_a.
Identification code 	1e_a
Empirical formula 	Al2 Li2 O12 Si4
Formula weight 	372.20
Temperature 	100(2) K
Wavelength 	0.7288 Å
Crystal system 	Monoclinic
Space group 	C2/c
Unit cell dimensions	a = 9.4667(9) Å	a= 90°.
	b = 8.3854(8) Å	b= 110.211(3)°.
	c = 5.2222(5) Å	g = 90°.
Volume	389.02(6) Å3
Z	2
Density (calculated)	3.177 Mg/m3
Absorption coefficient	1.139 mm-1
F(000)	368
Crystal size	0.160 x 0.050 x 0.040 mm3
Theta range for data collection	3.426 to 36.572°.
Index ranges	-15<=h<=15, -13<=k<=13, -8<=l<=8
Reflections collected	7479
Independent reflections	863 [R(int) = 0.0263]
Completeness to theta = 25.930°	98.3 % 
Absorption correction	Semi-empirical from equivalents
Max. and min. transmission	0.956 and 0.899
Refinement method	Full-matrix least-squares on F2
Data / restraints / parameters	863 / 0 / 47
Goodness-of-fit on F2	1.143
Final R indices [I>2sigma(I)]	R1 = 0.0199, wR2 = 0.0617
R indices (all data)	R1 = 0.0208, wR2 = 0.0627
Extinction coefficient	n/a
Largest diff. peak and hole	0.427 and -0.582 e.Å-3

 Table 2.  Atomic coordinates  ( x 104) and equivalent  isotropic displacement parameters (Å2x 103)
for 1e_a.  U(eq) is defined as one third of  the trace of the orthogonalized Uij tensor.
________________________________________________________________________________ 
	x	y	z	U(eq)
________________________________________________________________________________  
Si(1)	7060(1)	4064(1)	2432(1)	2(1)
Al(2)	5000	929(1)	2500	2(1)
O(3)	6434(1)	5143(1)	4395(1)	4(1)
O(4)	8904(1)	4174(1)	3591(1)	3(1)
O(5)	6354(1)	2325(1)	1999(1)	4(1)
Li(6)	10000	2260(2)	2500	8(1)
________________________________________________________________________________ 
 Table 3.   Bond lengths [Å] and angles [°] for  1e_a.
_____________________________________________________ 
Si(1)-O(5) 	1.5879(5)
Si(1)-O(3) 	1.6252(5)
Si(1)-O(3)#1 	1.6303(5)
Si(1)-O(4) 	1.6410(6)
Si(1)-Li(6)#2 	2.8633(8)
Si(1)-Li(6) 	3.1572(10)
Al(2)-O(5)#3 	1.8198(5)
Al(2)-O(5) 	1.8199(5)
Al(2)-O(4)#4 	1.9463(5)
Al(2)-O(4)#5 	1.9463(5)
Al(2)-O(4)#6 	1.9935(5)
Al(2)-O(4)#7 	1.9935(5)
Al(2)-Li(6)#5 	3.0205(10)
Al(2)-Li(6)#2 	3.0205(10)
Al(2)-Li(6)#6 	3.076(2)
O(3)-Li(6)#8 	2.2462(17)
O(4)-Li(6) 	2.0938(16)
O(5)-Li(6)#2 	2.2812(6)

O(5)-Si(1)-O(3)	111.93(3)
O(5)-Si(1)-O(3)#1	104.03(3)
O(3)-Si(1)-O(3)#1	107.31(2)
O(5)-Si(1)-O(4)	116.49(3)
O(3)-Si(1)-O(4)	108.03(3)
O(3)#1-Si(1)-O(4)	108.60(3)
O(5)-Si(1)-Li(6)#2	52.65(4)
O(3)-Si(1)-Li(6)#2	119.99(3)
O(3)#1-Si(1)-Li(6)#2	51.50(4)
O(4)-Si(1)-Li(6)#2	131.36(2)
O(5)-Si(1)-Li(6)	83.56(4)
O(3)-Si(1)-Li(6)	140.52(2)
O(3)#1-Si(1)-Li(6)	103.25(2)
O(4)-Si(1)-Li(6)	36.99(3)
Li(6)#2-Si(1)-Li(6)	98.518(10)
O(5)#3-Al(2)-O(5)	99.93(3)
O(5)#3-Al(2)-O(4)#4	91.87(2)
O(5)-Al(2)-O(4)#4	91.40(2)
O(5)#3-Al(2)-O(4)#5	91.40(2)
O(5)-Al(2)-O(4)#5	91.87(2)
O(4)#4-Al(2)-O(4)#5	174.91(3)
O(5)#3-Al(2)-O(4)#6	88.40(2)
O(5)-Al(2)-O(4)#6	167.83(2)
O(4)#4-Al(2)-O(4)#6	97.21(2)
O(4)#5-Al(2)-O(4)#6	78.98(2)
O(5)#3-Al(2)-O(4)#7	167.83(2)
O(5)-Al(2)-O(4)#7	88.40(2)
O(4)#4-Al(2)-O(4)#7	78.98(2)
O(4)#5-Al(2)-O(4)#7	97.21(2)
O(4)#6-Al(2)-O(4)#7	84.85(3)
O(5)#3-Al(2)-Li(6)#5	48.87(2)
O(5)-Al(2)-Li(6)#5	90.64(3)
O(4)#4-Al(2)-Li(6)#5	140.34(2)
O(4)#5-Al(2)-Li(6)#5	43.51(3)
O(4)#6-Al(2)-Li(6)#5	88.21(3)
O(4)#7-Al(2)-Li(6)#5	140.67(3)
O(5)#3-Al(2)-Li(6)#2	90.63(3)
O(5)-Al(2)-Li(6)#2	48.87(2)
O(4)#4-Al(2)-Li(6)#2	43.51(3)
O(4)#5-Al(2)-Li(6)#2	140.34(2)
O(4)#6-Al(2)-Li(6)#2	140.67(3)
O(4)#7-Al(2)-Li(6)#2	88.21(3)
Li(6)#5-Al(2)-Li(6)#2	119.64(7)
O(5)#3-Al(2)-Li(6)#6	130.033(17)
O(5)-Al(2)-Li(6)#6	130.033(17)
O(4)#4-Al(2)-Li(6)#6	87.457(16)
O(4)#5-Al(2)-Li(6)#6	87.457(16)
O(4)#6-Al(2)-Li(6)#6	42.424(16)
O(4)#7-Al(2)-Li(6)#6	42.424(16)
Li(6)#5-Al(2)-Li(6)#6	120.18(3)
Li(6)#2-Al(2)-Li(6)#6	120.18(3)
Si(1)-O(3)-Si(1)#9	138.70(3)
Si(1)-O(3)-Li(6)#8	117.29(3)
Si(1)#9-O(3)-Li(6)#8	93.88(3)
Si(1)-O(4)-Al(2)#5	119.87(3)
Si(1)-O(4)-Al(2)#10	121.89(3)
Al(2)#5-O(4)-Al(2)#10	101.02(2)
Si(1)-O(4)-Li(6)	114.87(4)
Al(2)#5-O(4)-Li(6)	96.70(2)
Al(2)#10-O(4)-Li(6)	97.61(4)
Si(1)-O(5)-Al(2)	148.57(3)
Si(1)-O(5)-Li(6)#2	93.75(5)
Al(2)-O(5)-Li(6)#2	94.20(4)
O(4)#11-Li(6)-O(4)	79.93(8)
O(4)#11-Li(6)-O(3)#7	140.06(2)
O(4)-Li(6)-O(3)#7	116.41(2)
O(4)#11-Li(6)-O(3)#12	116.41(2)
O(4)-Li(6)-O(3)#12	140.06(2)
O(3)#7-Li(6)-O(3)#12	75.56(7)
O(4)#11-Li(6)-O(5)#13	90.49(5)
O(4)-Li(6)-O(5)#13	75.98(4)
O(3)#7-Li(6)-O(5)#13	127.83(7)
O(3)#12-Li(6)-O(5)#13	68.15(3)
O(4)#11-Li(6)-O(5)#2	75.98(4)
O(4)-Li(6)-O(5)#2	90.49(5)
O(3)#7-Li(6)-O(5)#2	68.15(3)
O(3)#12-Li(6)-O(5)#2	127.83(7)
O(5)#13-Li(6)-O(5)#2	162.46(10)
O(4)#11-Li(6)-Si(1)#2	107.46(2)
O(4)-Li(6)-Si(1)#2	107.13(2)
O(3)#7-Li(6)-Si(1)#2	34.615(16)
O(3)#12-Li(6)-Si(1)#2	102.11(7)
O(5)#13-Li(6)-Si(1)#2	162.04(7)
O(5)#2-Li(6)-Si(1)#2	33.598(18)
O(4)#11-Li(6)-Si(1)#13	107.13(2)
O(4)-Li(6)-Si(1)#13	107.46(2)
O(3)#7-Li(6)-Si(1)#13	102.11(7)
O(3)#12-Li(6)-Si(1)#13	34.615(17)
O(5)#13-Li(6)-Si(1)#13	33.598(18)
O(5)#2-Li(6)-Si(1)#13	162.04(7)
Si(1)#2-Li(6)-Si(1)#13	134.35(8)
O(4)#11-Li(6)-Al(2)#5	89.88(6)
O(4)-Li(6)-Al(2)#5	39.79(2)
O(3)#7-Li(6)-Al(2)#5	126.71(2)
O(3)#12-Li(6)-Al(2)#5	101.356(16)
O(5)#13-Li(6)-Al(2)#5	36.93(2)
O(5)#2-Li(6)-Al(2)#5	130.24(6)
Si(1)#2-Li(6)-Al(2)#5	140.274(15)
Si(1)#13-Li(6)-Al(2)#5	67.722(10)
O(4)#11-Li(6)-Al(2)#2	39.79(2)
O(4)-Li(6)-Al(2)#2	89.88(6)
O(3)#7-Li(6)-Al(2)#2	101.356(16)
O(3)#12-Li(6)-Al(2)#2	126.71(2)
O(5)#13-Li(6)-Al(2)#2	130.24(6)
O(5)#2-Li(6)-Al(2)#2	36.93(2)
Si(1)#2-Li(6)-Al(2)#2	67.722(10)
Si(1)#13-Li(6)-Al(2)#2	140.274(15)
Al(2)#5-Li(6)-Al(2)#2	119.64(7)
O(4)#11-Li(6)-Al(2)#10	39.96(4)
O(4)-Li(6)-Al(2)#10	39.96(4)
O(3)#7-Li(6)-Al(2)#10	142.22(3)
O(3)#12-Li(6)-Al(2)#10	142.22(3)
O(5)#13-Li(6)-Al(2)#10	81.23(5)
O(5)#2-Li(6)-Al(2)#10	81.23(5)
Si(1)#2-Li(6)-Al(2)#10	112.82(4)
Si(1)#13-Li(6)-Al(2)#10	112.82(4)
Al(2)#5-Li(6)-Al(2)#10	59.82(3)
Al(2)#2-Li(6)-Al(2)#10	59.82(3)
O(4)#11-Li(6)-Si(1)#11	28.136(14)
O(4)-Li(6)-Si(1)#11	98.47(7)
O(3)#7-Li(6)-Si(1)#11	141.25(4)
O(3)#12-Li(6)-Si(1)#11	88.713(14)
O(5)#13-Li(6)-Si(1)#11	74.69(3)
O(5)#2-Li(6)-Si(1)#11	96.77(4)
Si(1)#2-Li(6)-Si(1)#11	121.323(11)
Si(1)#13-Li(6)-Si(1)#11	81.482(10)
Al(2)#5-Li(6)-Si(1)#11	90.72(4)
Al(2)#2-Li(6)-Si(1)#11	60.37(2)
Al(2)#10-Li(6)-Si(1)#11	61.37(3)
_____________________________________________________________ 
Symmetry transformations used to generate equivalent atoms: 
#1 x,-y+1,z-1/2    #2 -x+3/2,-y+1/2,-z    #3 -x+1,y,-z+1/2      
#4 x-1/2,-y+1/2,z-1/2    #5 -x+3/2,-y+1/2,-z+1      
#6 x-1/2,y-1/2,z    #7 -x+3/2,y-1/2,-z+1/2    #8 x-1/2,y+1/2,z      
#9 x,-y+1,z+1/2    #10 x+1/2,y+1/2,z    #11 -x+2,y,-z+1/2      
#12 x+1/2,y-1/2,z    #13 x+1/2,-y+1/2,z+1/2      

 Table 4.   Anisotropic displacement parameters  (Å2x 103) for 1e_a.  The anisotropic
displacement factor exponent takes the form:  -2p2[ h2 a*2U11 + ...  + 2 h k a* b* U12 ]
______________________________________________________________________________ 
	U11	U22 	U33	U23	U13	U12
______________________________________________________________________________ 
Si(1)	2(1) 	2(1)	2(1) 	0(1)	0(1) 	0(1)
Al(2)	2(1) 	2(1)	2(1) 	0	0(1) 	0
O(3)	3(1) 	5(1)	3(1) 	-1(1)	1(1) 	0(1)
O(4)	2(1) 	3(1)	3(1) 	0(1)	0(1) 	0(1)
O(5)	4(1) 	3(1)	5(1) 	0(1)	2(1) 	-1(1)
Li(6)	8(1) 	7(1)	8(1) 	0	3(1) 	0
______________________________________________________________________________ 
 
 
