# Supplementary material for: Defect-Mediated Diffusion Pathways in Spodumene Accelerate Lithium Transport
Source: ACS Mater Lett. 2025 Sep 8;7(10):3388–93. doi: 10.1021/acsmaterialslett.5c00876 (PMC12505375; doi:10.1021/acsmaterialslett.5c00876)
Supplement: Supplementary file 3 [file tz5c00876_si_003.zip › 1g_sample3/1g_a.rtf]

  Table 1.  Crystal data and structure refinement for 1g_a.
Identification code 	1g_a
Empirical formula 	Al Li O6 Si2
Formula weight 	186.10
Temperature 	100(2) K
Wavelength 	0.7288 Å
Crystal system 	Monoclinic
Space group 	C2/c
Unit cell dimensions	a = 9.4680(9) Å	a= 90°.
	b = 8.3860(8) Å	b= 110.196(3)°.
	c = 5.2262(5) Å	g = 90°.
Volume	389.44(6) Å3
Z	4
Density (calculated)	3.174 Mg/m3
Absorption coefficient	1.137 mm-1
F(000)	368
Crystal size	0.110 x 0.040 x 0.040 mm3
Theta range for data collection	3.425 to 36.565°.
Index ranges	-14<=h<=15, -13<=k<=13, -8<=l<=8
Reflections collected	7363
Independent reflections	868 [R(int) = 0.0356]
Completeness to theta = 25.930°	98.9 % 
Absorption correction	Semi-empirical from equivalents
Max. and min. transmission	0.956 and 0.869
Refinement method	Full-matrix least-squares on F2
Data / restraints / parameters	868 / 0 / 47
Goodness-of-fit on F2	1.097
Final R indices [I>2sigma(I)]	R1 = 0.0239, wR2 = 0.0700
R indices (all data)	R1 = 0.0255, wR2 = 0.0711
Extinction coefficient	n/a
Largest diff. peak and hole	0.625 and -0.711 e.Å-3

 Table 2.  Atomic coordinates  ( x 104) and equivalent  isotropic displacement parameters (Å2x 103)
for 1g_a.  U(eq) is defined as one third of  the trace of the orthogonalized Uij tensor.
________________________________________________________________________________ 
	x	y	z	U(eq)
________________________________________________________________________________  
Si(1)	7060(1)	4065(1)	2432(1)	2(1)
Al(2)	5000	930(1)	2500	2(1)
O(3)	6434(1)	5141(1)	4398(1)	4(1)
O(4)	8904(1)	4175(1)	3590(1)	3(1)
O(5)	6354(1)	2325(1)	1997(1)	4(1)
Li(6)	10000	2250(3)	2500	5(1)
________________________________________________________________________________ 
 Table 3.   Bond lengths [Å] and angles [°] for  1g_a.
_____________________________________________________ 
Si(1)-O(5) 	1.5879(7)
Si(1)-O(3) 	1.6255(7)
Si(1)-O(3)#1 	1.6309(7)
Si(1)-O(4) 	1.6414(7)
Si(1)-Li(6)#2 	2.8621(10)
Si(1)-Li(6) 	3.1618(12)
Al(2)-O(5)#3 	1.8206(7)
Al(2)-O(5) 	1.8206(7)
Al(2)-O(4)#4 	1.9483(7)
Al(2)-O(4)#5 	1.9483(7)
Al(2)-O(4)#6 	1.9936(7)
Al(2)-O(4)#7 	1.9936(7)
Al(2)-Li(6)#5 	3.0262(12)
Al(2)-Li(6)#2 	3.0262(12)
Al(2)-Li(6)#6 	3.085(3)
O(3)-Li(6)#8 	2.242(2)
O(4)-Li(6) 	2.101(2)
O(5)-Li(6)#2 	2.2834(8)

O(5)-Si(1)-O(3)	111.91(4)
O(5)-Si(1)-O(3)#1	104.02(4)
O(3)-Si(1)-O(3)#1	107.36(3)
O(5)-Si(1)-O(4)	116.49(4)
O(3)-Si(1)-O(4)	108.01(3)
O(3)#1-Si(1)-O(4)	108.59(3)
O(5)-Si(1)-Li(6)#2	52.77(5)
O(3)-Si(1)-Li(6)#2	119.96(3)
O(3)#1-Si(1)-Li(6)#2	51.38(5)
O(4)-Si(1)-Li(6)#2	131.39(3)
O(5)-Si(1)-Li(6)	83.40(4)
O(3)-Si(1)-Li(6)	140.51(3)
O(3)#1-Si(1)-Li(6)	103.32(3)
O(4)-Si(1)-Li(6)	37.11(4)
Li(6)#2-Si(1)-Li(6)	98.530(11)
O(5)#3-Al(2)-O(5)	99.98(4)
O(5)#3-Al(2)-O(4)#4	91.93(3)
O(5)-Al(2)-O(4)#4	91.38(3)
O(5)#3-Al(2)-O(4)#5	91.38(3)
O(5)-Al(2)-O(4)#5	91.93(3)
O(4)#4-Al(2)-O(4)#5	174.85(4)
O(5)#3-Al(2)-O(4)#6	88.37(3)
O(5)-Al(2)-O(4)#6	167.84(3)
O(4)#4-Al(2)-O(4)#6	97.20(3)
O(4)#5-Al(2)-O(4)#6	78.95(3)
O(5)#3-Al(2)-O(4)#7	167.84(3)
O(5)-Al(2)-O(4)#7	88.37(3)
O(4)#4-Al(2)-O(4)#7	78.95(3)
O(4)#5-Al(2)-O(4)#7	97.20(3)
O(4)#6-Al(2)-O(4)#7	84.85(4)
O(5)#3-Al(2)-Li(6)#5	48.80(3)
O(5)-Al(2)-Li(6)#5	90.59(4)
O(4)#4-Al(2)-Li(6)#5	140.32(3)
O(4)#5-Al(2)-Li(6)#5	43.59(3)
O(4)#6-Al(2)-Li(6)#5	88.31(4)
O(4)#7-Al(2)-Li(6)#5	140.73(3)
O(5)#3-Al(2)-Li(6)#2	90.59(4)
O(5)-Al(2)-Li(6)#2	48.80(3)
O(4)#4-Al(2)-Li(6)#2	43.59(3)
O(4)#5-Al(2)-Li(6)#2	140.32(3)
O(4)#6-Al(2)-Li(6)#2	140.73(3)
O(4)#7-Al(2)-Li(6)#2	88.31(4)
Li(6)#5-Al(2)-Li(6)#2	119.42(8)
O(5)#3-Al(2)-Li(6)#6	130.01(2)
O(5)-Al(2)-Li(6)#6	130.01(2)
O(4)#4-Al(2)-Li(6)#6	87.43(2)
O(4)#5-Al(2)-Li(6)#6	87.43(2)
O(4)#6-Al(2)-Li(6)#6	42.43(2)
O(4)#7-Al(2)-Li(6)#6	42.43(2)
Li(6)#5-Al(2)-Li(6)#6	120.29(4)
Li(6)#2-Al(2)-Li(6)#6	120.29(4)
Si(1)-O(3)-Si(1)#9	138.75(4)
Si(1)-O(3)-Li(6)#8	117.12(4)
Si(1)#9-O(3)-Li(6)#8	93.98(4)
Si(1)-O(4)-Al(2)#5	119.86(4)
Si(1)-O(4)-Al(2)#10	121.90(4)
Al(2)#5-O(4)-Al(2)#10	101.05(3)
Si(1)-O(4)-Li(6)	114.76(5)
Al(2)#5-O(4)-Li(6)	96.65(3)
Al(2)#10-O(4)-Li(6)	97.77(5)
Si(1)-O(5)-Al(2)	148.49(5)
Si(1)-O(5)-Li(6)#2	93.61(6)
Al(2)-O(5)-Li(6)#2	94.34(5)
O(4)#11-Li(6)-O(4)	79.61(9)
O(4)#11-Li(6)-O(3)#7	140.04(3)
O(4)-Li(6)-O(3)#7	116.47(3)
O(4)#11-Li(6)-O(3)#12	116.47(3)
O(4)-Li(6)-O(3)#12	140.04(3)
O(3)#7-Li(6)-O(3)#12	75.82(8)
O(4)#11-Li(6)-O(5)#13	90.29(6)
O(4)-Li(6)-O(5)#13	75.84(5)
O(3)#7-Li(6)-O(5)#13	128.13(8)
O(3)#12-Li(6)-O(5)#13	68.20(3)
O(4)#11-Li(6)-O(5)#2	75.84(5)
O(4)-Li(6)-O(5)#2	90.29(6)
O(3)#7-Li(6)-O(5)#2	68.20(3)
O(3)#12-Li(6)-O(5)#2	128.13(8)
O(5)#13-Li(6)-O(5)#2	162.07(12)
O(4)#11-Li(6)-Si(1)#2	107.37(3)
O(4)-Li(6)-Si(1)#2	107.06(3)
O(3)#7-Li(6)-Si(1)#2	34.64(2)
O(3)#12-Li(6)-Si(1)#2	102.40(8)
O(5)#13-Li(6)-Si(1)#2	162.33(8)
O(5)#2-Li(6)-Si(1)#2	33.62(2)
O(4)#11-Li(6)-Si(1)#13	107.06(3)
O(4)-Li(6)-Si(1)#13	107.37(3)
O(3)#7-Li(6)-Si(1)#13	102.40(8)
O(3)#12-Li(6)-Si(1)#13	34.64(2)
O(5)#13-Li(6)-Si(1)#13	33.62(2)
O(5)#2-Li(6)-Si(1)#13	162.33(8)
Si(1)#2-Li(6)-Si(1)#13	134.67(9)
O(4)#11-Li(6)-Al(2)#5	89.65(7)
O(4)-Li(6)-Al(2)#5	39.75(3)
O(3)#7-Li(6)-Al(2)#5	126.83(3)
O(3)#12-Li(6)-Al(2)#5	101.33(2)
O(5)#13-Li(6)-Al(2)#5	36.86(3)
O(5)#2-Li(6)-Al(2)#5	130.01(8)
Si(1)#2-Li(6)-Al(2)#5	140.243(18)
Si(1)#13-Li(6)-Al(2)#5	67.662(11)
O(4)#11-Li(6)-Al(2)#2	39.75(3)
O(4)-Li(6)-Al(2)#2	89.65(7)
O(3)#7-Li(6)-Al(2)#2	101.33(2)
O(3)#12-Li(6)-Al(2)#2	126.83(3)
O(5)#13-Li(6)-Al(2)#2	130.01(8)
O(5)#2-Li(6)-Al(2)#2	36.86(3)
Si(1)#2-Li(6)-Al(2)#2	67.662(11)
Si(1)#13-Li(6)-Al(2)#2	140.243(18)
Al(2)#5-Li(6)-Al(2)#2	119.42(8)
O(4)#11-Li(6)-Al(2)#10	39.81(5)
O(4)-Li(6)-Al(2)#10	39.81(5)
O(3)#7-Li(6)-Al(2)#10	142.09(4)
O(3)#12-Li(6)-Al(2)#10	142.09(4)
O(5)#13-Li(6)-Al(2)#10	81.03(6)
O(5)#2-Li(6)-Al(2)#10	81.03(6)
Si(1)#2-Li(6)-Al(2)#10	112.66(5)
Si(1)#13-Li(6)-Al(2)#10	112.66(5)
Al(2)#5-Li(6)-Al(2)#10	59.71(4)
Al(2)#2-Li(6)-Al(2)#10	59.71(4)
O(4)#11-Li(6)-Si(1)#11	28.127(19)
O(4)-Li(6)-Si(1)#11	98.19(8)
O(3)#7-Li(6)-Si(1)#11	141.36(5)
O(3)#12-Li(6)-Si(1)#11	88.755(18)
O(5)#13-Li(6)-Si(1)#11	74.58(3)
O(5)#2-Li(6)-Si(1)#11	96.65(4)
Si(1)#2-Li(6)-Si(1)#11	121.278(13)
Si(1)#13-Li(6)-Si(1)#11	81.470(11)
Al(2)#5-Li(6)-Si(1)#11	90.57(4)
Al(2)#2-Li(6)-Si(1)#11	60.30(3)
Al(2)#10-Li(6)-Si(1)#11	61.23(4)
_____________________________________________________________ 
Symmetry transformations used to generate equivalent atoms: 
#1 x,-y+1,z-1/2    #2 -x+3/2,-y+1/2,-z    #3 -x+1,y,-z+1/2      
#4 x-1/2,-y+1/2,z-1/2    #5 -x+3/2,-y+1/2,-z+1      
#6 x-1/2,y-1/2,z    #7 -x+3/2,y-1/2,-z+1/2    #8 x-1/2,y+1/2,z      
#9 x,-y+1,z+1/2    #10 x+1/2,y+1/2,z    #11 -x+2,y,-z+1/2      
#12 x+1/2,y-1/2,z    #13 x+1/2,-y+1/2,z+1/2      

 Table 4.   Anisotropic displacement parameters  (Å2x 103) for 1g_a.  The anisotropic
displacement factor exponent takes the form:  -2p2[ h2 a*2U11 + ...  + 2 h k a* b* U12 ]
______________________________________________________________________________ 
	U11	U22 	U33	U23	U13	U12
______________________________________________________________________________ 
Si(1)	2(1) 	2(1)	2(1) 	0(1)	0(1) 	0(1)
Al(2)	2(1) 	2(1)	2(1) 	0	0(1) 	0
O(3)	3(1) 	5(1)	3(1) 	-2(1)	1(1) 	0(1)
O(4)	2(1) 	4(1)	3(1) 	0(1)	1(1) 	0(1)
O(5)	4(1) 	3(1)	5(1) 	0(1)	2(1) 	-2(1)
Li(6)	6(1) 	5(1)	5(1) 	0	1(1) 	0
______________________________________________________________________________ 
 
 
